# Differential Activation of Acid Sphingomyelinase and Ceramide Release Determines Invasiveness of *Neisseria meningitidis* into Brain Endothelial Cells

**DOI:** 10.1371/journal.ppat.1004160

**Published:** 2014-06-12

**Authors:** Alexander Simonis, Sabrina Hebling, Erich Gulbins, Sibylle Schneider-Schaulies, Alexandra Schubert-Unkmeir

**Affiliations:** 1 Institute of Hygiene and Microbiology, University of Wuerzburg, Wuerzburg, Germany; 2 Department of Molecular Medicine, University of Essen, Essen, Germany; 3 Institute for Virology and Immunbiology, University of Wuerzburg, Wuerzburg, Germany; University of Oxford, United Kingdom

## Abstract

The interaction with brain endothelial cells is central to the pathogenicity of *Neisseria meningitidis* infections. Here, we show that *N. meningitidis* causes transient activation of acid sphingomyelinase (ASM) followed by ceramide release in brain endothelial cells. In response to *N. meningitidis* infection, ASM and ceramide are displayed at the outer leaflet of the cell membrane and condense into large membrane platforms which also concentrate the ErbB2 receptor. The outer membrane protein Opc and phosphatidylcholine-specific phospholipase C that is activated upon binding of the pathogen to heparan sulfate proteoglycans, are required for *N. meningitidis*-mediated ASM activation. Pharmacologic or genetic ablation of ASM abrogated meningococcal internalization without affecting bacterial adherence. In accordance, the restricted invasiveness of a defined set of pathogenic isolates of the ST-11/ST-8 clonal complex into brain endothelial cells directly correlated with their restricted ability to induce ASM and ceramide release. In conclusion, ASM activation and ceramide release are essential for internalization of Opc-expressing meningococci into brain endothelial cells, and this segregates with invasiveness of *N. meningitidis* strains.

## Author Summary

*Neisseria meningitidis*, an obligate human pathogen, is a causative agent of septicemia and meningitis worldwide. Meningococcal infection manifests in a variety of forms, including meningitis, meningococcemia with meningitis or meningococcemia without obvious meningitis. The interaction of *N. meningitidis* with human cells lining the blood vessels of the blood-cerebrospinal fluid barrier is a prerequisite for the development of meningitis. As a major pathogenicity factor, the meningococcal outer membrane protein Opc enhances bacterial entry into brain endothelial cells, however, mechanisms underlying trapping of receptors and signaling molecules following this interaction remained elusive. We now show that Opc-expressing meningococci activate acid sphingomyelinase (ASM) in brain endothelial cells, which hydrolyses sphingomyelin to cause ceramide release and formation of extended ceramide-enriched membrane platforms wherein ErbB2, an important receptor involved in bacterial uptake, clusters. Mechanistically, ASM activation relied on binding of *N. meningitidis* to its attachment receptor, HSPG, followed by activation of PC-PLC. Meningococcal isolates of the ST-11 clonal complex, which are reported to be more likely to cause severe sepsis, but rarely meningitis, barely invaded brain endothelial cells and revealed a highly restricted ability to induce ASM and ceramide release. Thus, our results unravel a differential activation of the ASM/ceramide system by the species *N. meningitidis* determining its invasiveness into brain endothelial cells.

## Introduction

*Neisseria meningitidis* (*Nm*, the meningococcus) is a frequently found asymptomatic colonizer of the upper respiratory tract, which under certain circumstances may penetrate the mucosal membrane, reach the bloodstream and cause septicemia and/or meningitis. During the course of infection *N. meningitidis* is capable to interact with a variety of human cells including epithelial as well as peripheral and brain microvascular endothelial cells [1], [2]. To mediate association with this wide range of host cells, meningococcci express a variety of adhesins and invasins, including type IV pili (TfP) [3]–[5], the outer membrane proteins Opa and Opc and a number of newly identified minor adhesion or adhesion-like proteins [6]–[12]. As an important pathogenicity factor the integral outer membrane protein (OMP) Opc is particularly implicated in host cell invasion of endothelial cells [1], [8], [13], [14]. Opc is a beta barrel protein with five surface loops encoded by a single gene (*opcA*) and is antigenically stable [15], [16]. Opc expression is controlled at the transcriptional level by the length of a polycytidine stretch within its promoter region [17]. Opc is expressed by several virulent *N. meningitidis* lineages, but is absent from certain epidemic clones (ET-37/ST-11 clonal complex (cc)) and a few random endemic isolates [18]. Two epidemiological studies reported outbreaks where meningococcal strains of the ST-11 cc tend to cause severe sepsis with fatal outcome, but rarely meningitis [19], [20].

For *N. meningitidis* uptake, Opc links the meningococcus to the extracellular matrix components and serum proteins vitronectin and fibronectin followed by binding to αvβ3 or α5β1-integrins and activation of phosphotyrosine signalling and cytoskeletal rearrangement [1], [2], [21]–[23]. As observed for human epithelial cells, Opc can also bind to heparin-like molecules and to cell surface heparan sulfate proteoglycans (HSPGs) [24], which can mediate receptor interaction (referred to as *cis*-activation) to induce signaling and subsequent internalization [25].

Adhesion of fully encapsulated meningococci to host cells is facilitated primarily by pili. Though OMPs are partially masked by the polysaccharide capsule, they also efficiently support adhesion and invasion to eukaryotic cells especially on cells of high receptor density as would be induced in inflammatory conditions and/or lateral receptor aggregation [26].

Sphingomyelin is a major component of the outer plasma membrane layer where, under certain conditions of stress or the induction of inflammatory cytokines, it is metabolized into ceramide and phosphorylcholine by the activity of the acid sphingomyelinase (ASM). Once activated, this enzyme translocates from its lysosomal intracellular storage to outer cell membrane where it is co-displayed with released ceramides [27]–[35]. Due to their biophysical properties, ceramide-enriched membrane domains fuse into extended ceramide-enriched platforms which span a few hundred nanometers to several micrometers [27], [29]. In addition to altering membrane fluidity and rigidity, ceramide-enriched platforms serve to sort and eventually concentrate membrane receptors and membrane proximal signaling components thereby amplifying cellular responses and signal transduction [36]. Their role in enhancement of pathogen uptake has been directly revealed for *N. gonorrhoeae*, *Pseudomonas aeruginosa*, as well as rhinoviruses and measles virus [37]–[41]. In addition, *Staphylococcus aureus* triggers the formation of ceramide-enriched membrane platforms for induction of apoptosis [42]. It is as yet unknown whether SMase activation and ceramide release relates to *N. meningitidis* uptake especially in its natural target cells.

In this study we now show that *N. meningitidis* induces ASM activation, ceramide release and formation of ceramide-enriched platforms proximal to attached bacteria within the outer layer of the membrane of brain endothelial cells. Ceramide-enriched platforms in turn serve to cluster the ErbB2 receptor underneath adherent bacteria. Opc and activation of phosphatidylcholine-specific phospholipase C (PC-PLC) downstream of HSPGs is critical for *N. meningitidis* ASM activation, which proved to be crucial for *N. meningitidis* uptake but not adhesion. Stressing the importance of ASM activation in *N. meningitidis* invasion and pathogenesis, a less invasive defined set of pathogenic isolates of the ST-11/ST-8 cc was substantially less capable of inducing ASM activation and formation of ceramide-enriched platforms.

## Results

### Exposure of *N. meningitidis* to host cells induces ASM activation, ceramide release and formation of ceramide-enriched platforms

**Figure 1:**
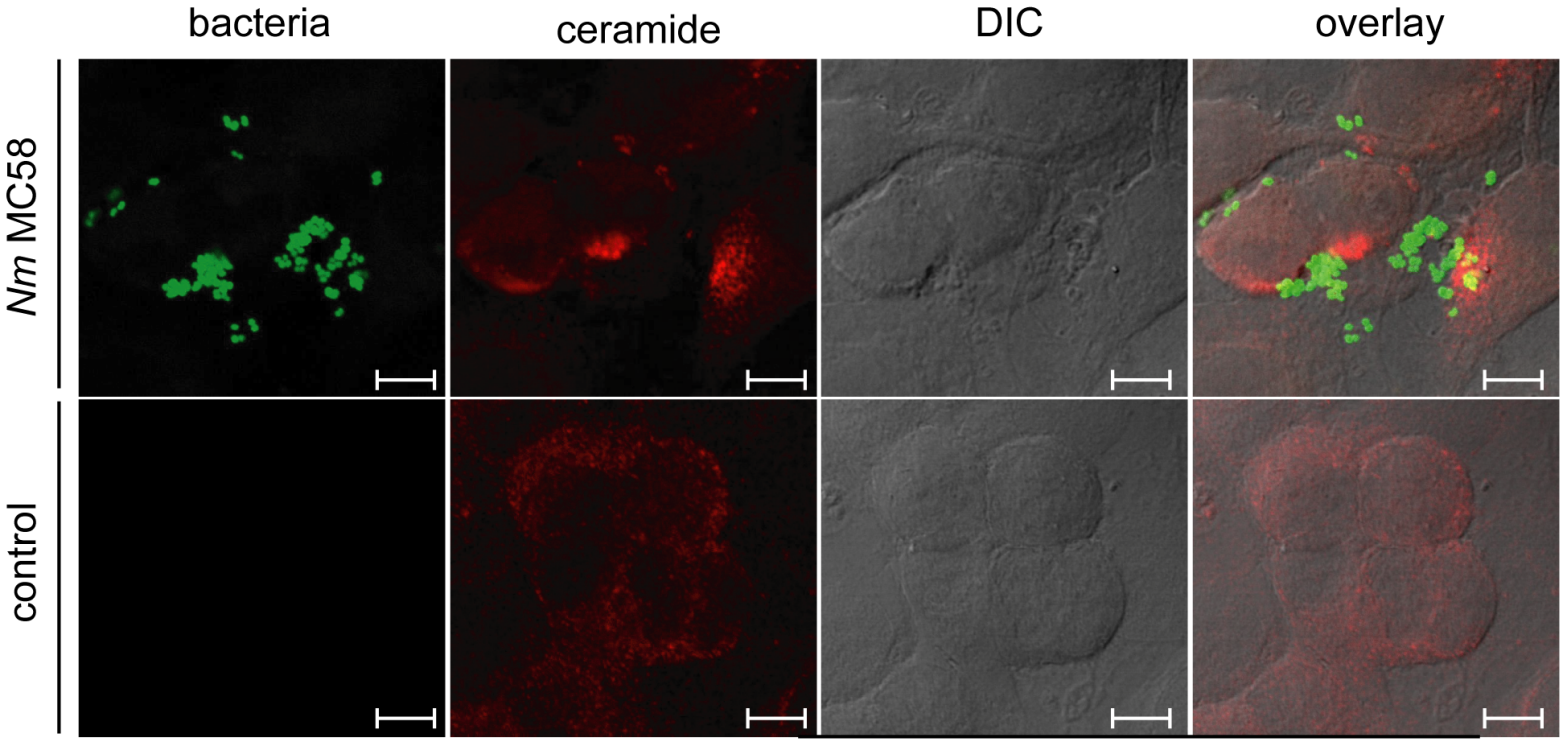
*N. meningitidis* induces the formation of ceramide-enriched membrane platforms on brain endothelial cells (HBMEC). HBMEC were infected with a GFP-expressing wildtype strain MC58 for 2 h (upper panels) or left uninfected (control cells, bottom panels), fixed, left intact, stained with anti-ceramide antibodies and secondary Cy3-conjugated anti-mouse-IgM antibodies and analyzed by confocal microscopy. Ceramides accumulate in close association with attached bacteria. The data are representative for 5 similar studies. Size bars represent 5 µm.

Because uptake of some pathogenic bacteria involved formation of ceramide-enriched membrane platforms [37]–[39], we investigated whether *N. meningitidis* employs a similar mechanism to infect and enter into eukaryotic cells. To analyse whether *N. meningitidis* stimulates surface display of ceramide on human brain microvascular endothelial cells (HBMEC), cells were infected with the GFP-expressing wildtype strain MC58 (ST-32 clonal complex (cc)), fixed and stained with an anti-ceramide antibody (mAb 15B4). *N. meningitidis* strain MC58 rapidly, but transiently induced formation of large extrafacial ceramide-enriched platforms, which reached a maximum within 2 hrs after infection (Fig. 1, Fig. S1) and decreased thereafter. Bacteria adhered to the cells within ceramide-enriched membrane platforms (Fig. 1A, upper panels). In unexposed control cells, shallow ceramide-specific signals were visible, but these were not condensed into large platforms (Fig. 1A, lower panels).

**Figure 2:**
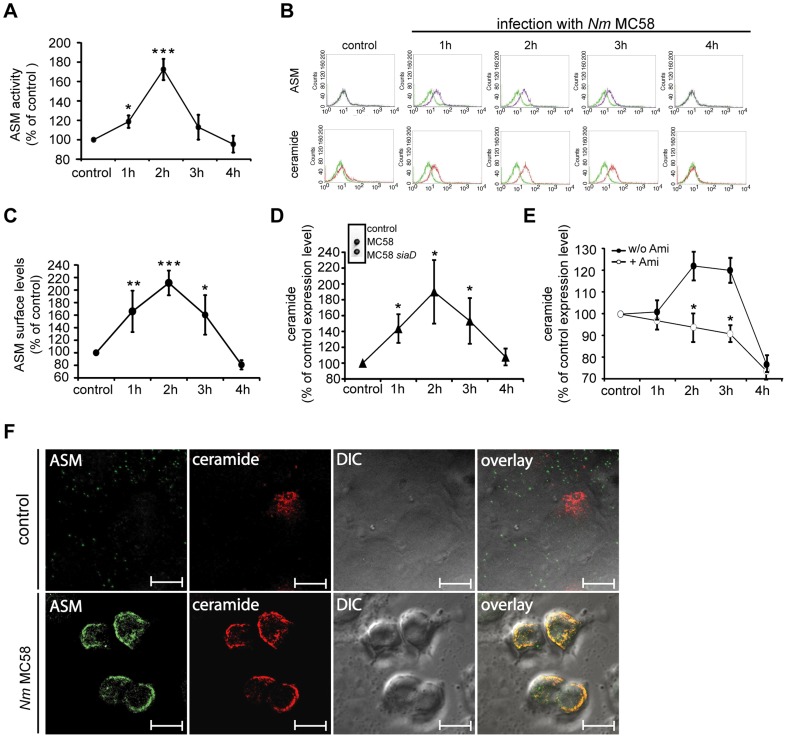
*N. meningitidis* infection activates ASM and causes membrane ceramide accumulation on HBMEC. (A) ASM activity was measured in whole cell lysates of HBMEC infected with *N. meningitidis* wildtype strain MC58 or not (control) after the indicated time intervals. Data (mean ± S.D.) were generated in three independent experiments, each performed in triplicate. (B to E) Surface ASM and ceramide levels on infected HBMEC were determined by flow cytometry analysis or spot assays (shown in Fig. 2D, insert). (B) Surface display of ASM (upper row, purple line) and ceramide (bottom row, red line) on HBMEC infected with *N. meningitidis* strain MC58 for 4 hrs, control, uninfected HBMEC. An isotype antibody served as a negative control in staining experiments (both rows, green line) (MFI of isotype control ceramide = 9.79±1.34; MFI of isotype control ASM = 13.12±0.53). (C) Surface ASM levels on infected HBMEC were determined in three independent experiments performed in duplicates. (D) Surface ceramide release on wildtype strain MC58 infected HBMEC (and, for control, uninfected cells) was determined by flow cytometry or spot assays (inset). An isotype antibody served as a negative control in staining experiments. For the spot assay, HBMEC were infected with wildtype strain MC58 and an isogenic, unencapsulated isolate MC58 *siaD* or were left uninfected (control) for 2 hrs. (E) Surface ceramide levels were estimated on HBMEC treated with amitriptyline (open circles) or not (filled circles) prior to infection (MFI of isotype control ceramide = 8.63±0.94). (F) Detection of ASM or ceramides on HBMEC infected with MC58 (lower panel) or left uninfected (control, upper panel) by microscopy. As representatively shown, ASM co-localized in ceramide-enriched membrane platforms on HBMEC upon infection with *N. meningitidis*. Size bars represent 10 µm. All data show mean values ± S.D. of three independent experiments done in duplicate. * *P*<0.05, ** *P*<0.01, *** *P*<0.001, relative to uninfected control cells or to cells infected without inhibitor. Fluorescence microscopy studies are representative for 3 similar studies.

Because surface accumulation of ceramides usually reflects acid sphingomyelinase (ASM) rather than neutral sphingomyelinase (NSM) activity, which acts on its substrate in the cytosolic layer, we directly assessed activation of these enzymes in response to *N. meningitidis* MC58 exposure directly in cell lysates or membrane preparations (for NSM), respectively. Mirroring the kinetics of extrafacial ceramide release (Fig. 1), *N. meningitidis* MC58 caused a rapid ASM activation, which peaked within 2 hrs and subsequently declined (Fig. 2 A). In contrast, NSM was not stimulated by *N. meningitidis* within this time frame (Fig. S2). The kinetics of ASM and ceramide surface display were monitored by flow cytometry (Fig. 2B–D) or by immunodetection of a ceramide-specific antibody bound to intact cells (Fig. 2D, inset) at various time intervals following *N. meningitidis* MC58 exposure. Irrespective of the method used, ASM and ceramide were readily detectable on the cell surface within 2 hrs and their expression declined thereafter. Pre-treatment of cells with the functional ASM inhibitor amitriptyline prevented surface release of ceramides after *N. meningitidis* MC58 exposure indicating that this process depends on ASM (Fig. 2E). Finally, while ASM was not and ceramide was barely detectable on the surface of uninfected HBMEC cells, both molecules were readily co-detected 2 hrs following exposure of the cells to MC58 (Fig. 2F). *N. meningitidis* MC58–induced ASM surface location and ceramide release were not restricted to HBMEC as revealed by experiments involving HBMEC/ciβ, which slightly differed with regard to the kinetics of peak levels (3 hrs rather than 2 hrs), but not efficiency of ceramide release (Fig. S3).

### *N. meningitidis* triggers ASM induction and ceramide release via activation of PC-PLC

**Figure 3:**
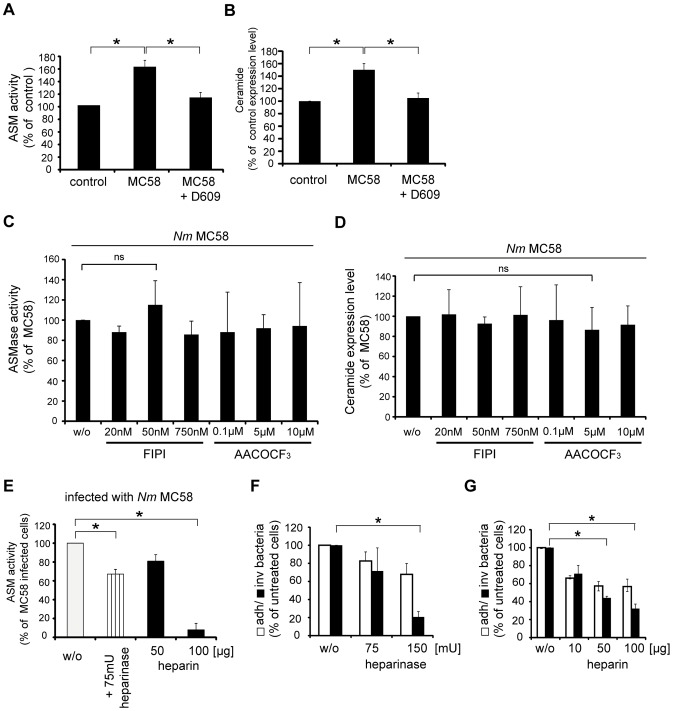
Phosphatidylcholine-specific phospholipase C (PC-PLC) is involved in ASM activation and bacterial uptake by HBMEC. HBMEC were treated with the PC-PLC inhibitor D609 (100 µM) 30 min prior to infection with *N. meningitidis* MC58 or left untreated, and (A) ASM activity and (B) ceramide surface display were determined 2 hrs after infection. Non-infected cells served as control. Results represent mean ± S.D. of three independent experiments done in triplicates. (C and D) HBMEC were treated with indicated concentrations of the PLA_2_ inhibitor AACOCF_3_ 30 min prior to infection with *N. meningitidis* MC58 or with indicated concentrations of the PLD inhibitor FIPI 1 h prior to infection and ASM activity (C) and ceramide (D) surface display were determined 2 hrs after infection. Results represent mean ± S.D. of three independent experiments done in duplicates and are shown in relation to ASM activity and ceramide expression in MC58-infected HBMEC. ns = not significant. (E) Cells were treated with 75 mU heparinase (striped bar) or heparin (black bars) prior to infection with *N. meningitidis* strain MC58, and ASM activity was assessed on these and unmodified infected cells (w/o, white bar) after 2 hrs. Results represent mean ± S.D. of three independent experiments done in triplicate. * *P*<0.05, relative to untreated cells. (F and G) Cells were pretreated with 75 mU and 150 mU heparinase or indicated concentration of heparin or left untreated (w/o) and infected with *N. meningitidis* strain MC58. Number of adherent (open bars) and invasive (filled bars) bacteria was determined by gentamicin protection assays 4 hrs after infection. The data show mean values ± S.D. of three independent experiments done in duplicate. * *P*<0.05, relative to cells infected without inhibitor.

Previous studies implicated a regulation of ASM by diacylglycerol (DAG) release in response to phosphatidylcholine-specific phospholipase C (PC-PLC) activity [43], [44]. This also applied to the related species, *N. gonorrhoeae*, where PC consumption by PC-PLC activity released DAG resulting in ASM activation [37]. PC-PLC activity was measured in lysates of HBMEC infected with *N. meningitidis* MC58 over time in the presence or absence of the PC-PLC inhibitor D609. *N. meningitidis* MC58 caused a significant PC-PLC activation, which peaked within 2 hrs, and this was inhibited by preincubation of HBMEC with D609 (Fig. S4). To rule out a possible role of PC-PLC also in *N. meningitidis* driven ASM activation, ASM activity was assessed in lysates of HBMEC that were treated with the PC-PLC inhibitor D609 prior to infection. D609 indeed blocked the activation of ASM during meningococcal infection (Fig. 3A). Moreover, cell surface ceramide release was significantly reduced in the presence of D609 as demonstrated by flow cytometry 2 hrs following infection with *N. meningitidis* MC58 (Fig. 3B). To address a potential contribution of phospholipase A_2_ (PLA_2_) or phospholipase D (PLD) in DAG generation for ASM activation, activity of the enzyme was assessed in lysates of HBMEC treated with PLA_2_ inhibitor AACOCF_3_ or PLD inhibitor 5-fluoro-2-indolyl des-chlorohalopemide (FIPI), respectively, prior to infection. Indicating that these enzymes are not involved in meningococcal ASM activation, these compounds did not affect ASM activity (Fig. 3C) nor cell surface ceramide display (Fig. 3D).

For *N. gonorrhoeae*, PC consumption by PC-PLC is initiated by binding of Opa-expressing strains to heparan sulphate proteoglycans (HSPGs) [37]. Opc is also able to mediate adhesion to and invasion of epithelial cells by binding to HSPGs [24]. To analyse whether PC-PLC and ASM are activated downstream of HSPGs, two different experimental approaches were taken: ASM activity was assessed in HBMEC infected in the presence of heparin that blocks possible Opc-HSPG interactions, or cells were treated with heparinase III, which cleaves heparan sulphate moieties, prior to infection. Both conditions significantly reduced ASM activation by *N. meningitidis* (Fig. 3E), along with meningococcal uptake into HBMEC (Fig. 3F and G), indicating an important role of this enzyme in this process.

### Acid sphingomyelinase activation supports uptake of *N. meningitidis*

**Figure 4:**
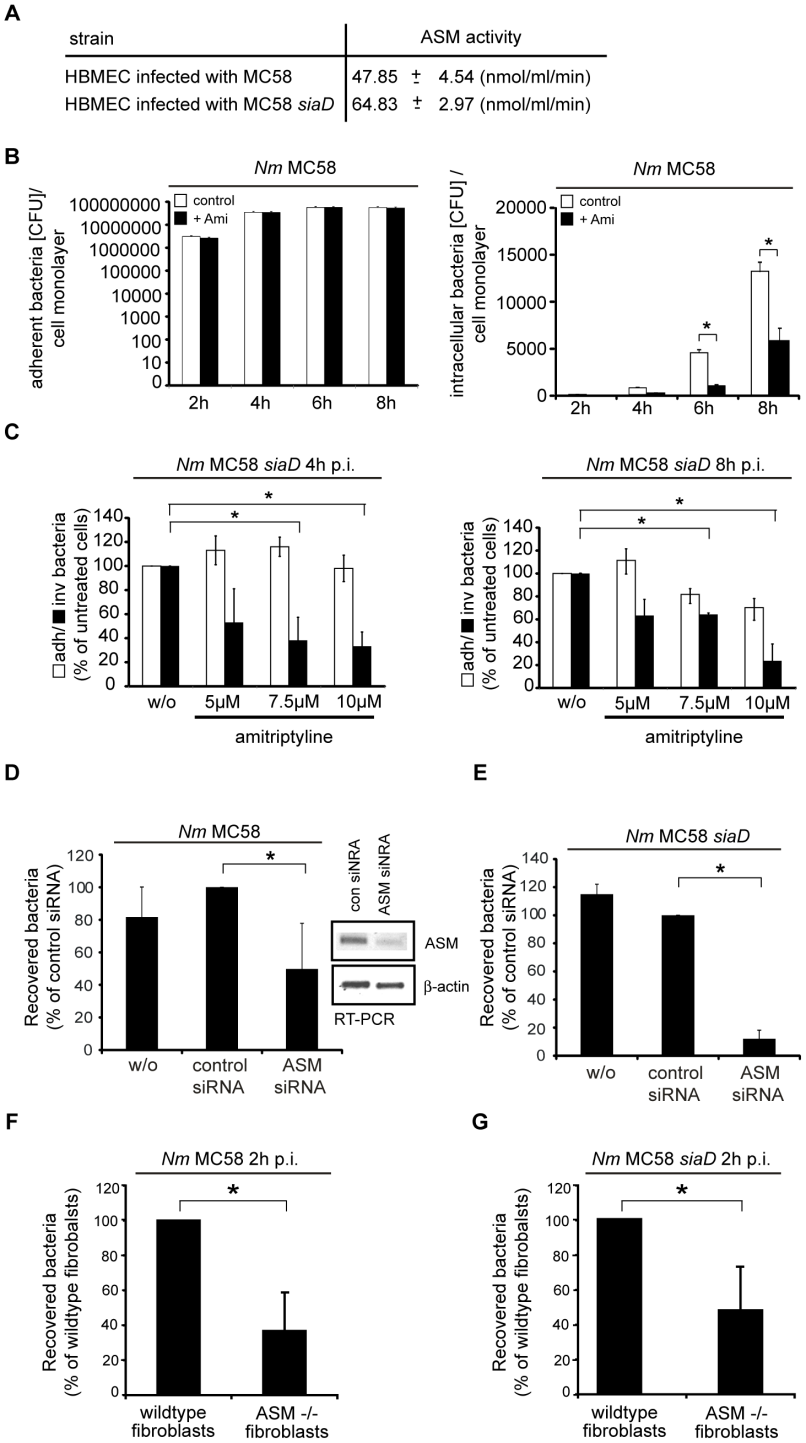
Inhibition of acid sphingomyelinase prevents *N. meningitidis* uptake by host cells. (A) ASM activity was determined in whole cell lysates of HBMEC infected with *N. meningitidis* wildtype strain MC58 or unencapsulated strain MC58 *siaD* for 2 h. Data are compiled from three independent experiments performed in triplicate. (B) Wildtype strain MC58 adherence on (left panel) and invasion into (right panel) HBMEC which were preincubated with 10 µM amitriptyline (+ Ami, filled bars) or solvent (DMSO) only (control, open bars) within the time interval indicated were determined by gentamicin protection assays. The data show mean values ± S.D. of three independent experiments done in duplicate. * *P*<0.05, relative to cells infected without inhibitor. (C) Adherence (white bars) and invasion (black bars) of the unencapsulated strain MC58 *siaD* were determined on HBMEC pre-incubated with solvent only (w/o) or amitriptyline 4 h (left panel) or 8 h (right panel) following infection. Data are relative values with solvent only set to 100 percent and show mean values ± S.D. of three independent experiments done in duplicate. (D and E) Intracellular bacteria were determined in untransfected cells (w/o) or in HBMEC transfected with ASM-specific or control siRNA (con siRNA) 72 hrs prior to infection with strain MC58 (D) and mutant strain MC58 *siaD* (E) for 4 h. The graph represents mean values ± S.D. of three independent experiments done in duplicate. * *P*<0.05, relative to cells transfected with the control siRNA. Genetic knockdown of ASM was determined in total RNA by RT-PCR analysis. Amplification of β-actin served as control. (F and G) Mouse embryonic fibroblasts (MEFs) of wildtype (wildype fibroblasts) or ASM-deficient (ASM^−/−^ MEFs) mice were infected with MC58 and MC58 *siaD* for 2 h. Intracellular bacteria were estimated at 2 h p.i. by gentamicin protection assays. The graph represents mean value ± S.D. of three different independent experiments done in duplicate. * *P*<0.05.

To analyze whether ASM activation was of functional importance in meningococcal uptake directly, HBMEC were treated with the ASM inhibitor amitriptyline 30 min prior to infection with *N. meningitidis* MC58 and an isogenic unencapsulated mutant strain MC58 *siaD*, and adhesion and invasion were determined over time. The unencapsulated mutant was included into these experiments due to its higher invasive capacity [1]. Interestingly, ASM activation induced by MC58 *siaD* after 2 h significantly exceeded that induced by wildtype MC58 (Fig. 4A). As determined in gentamicin protection assays, pharmacological ASM inhibition reduced intracellular accumulation of *N. meningitidis* MC58 (Fig. 4B, right panel) and MC58 *siaD* (Fig. 4C) (about 70% inhibition with 10 µM amitriptyline at 4 h p.i. [*P*<0.05] and about 80% inhibition at 8 h p.i. [*P*<0.05], dose-dependent data shown for MC58 *siaD* only) (Fig. 4C). ASM inhibition did, however, not affect adhesion of *N. meningitidis* to HBMEC as determined by the estimation of cell-adherent bacteria (Fig. 4B, left panel, for MC58, Fig. 4C for MC58 *siaD*) or bacterial growth (data not shown). We took two independent genetic approaches to verify the importance of the ASM for *N. meningitidis* invasion. Firstly, RNAi-mediated knockdown of ASM transcripts reduced *N. meningitidis* uptake by HBMEC by about 40% for MC58 and >90% for MC58 *siaD*, while pathogen uptake was not affected by a scrambled control siRNA (Fig. 4D and E). Secondly, we comparatively analyzed *N. meningitidis* uptake into fibroblasts derived from ASM^−/−^ or wild-type mouse embryos (MEFs). Wild-type MEFs internalised *N. meningitidis* with similar kinetics, but less efficiently (one log lower) than HBMEC, however, while ASM-deficient MEFs were significantly impaired in the ability to internalize *N. meningitidis* (Fig. 4F and G; later time points could not be analyzed due to toxicity of the infection in these cultures).

### ASM-deficient Niemann-Pick fibroblasts are significantly less invaded by *N. meningitidis*

**Figure 5:**
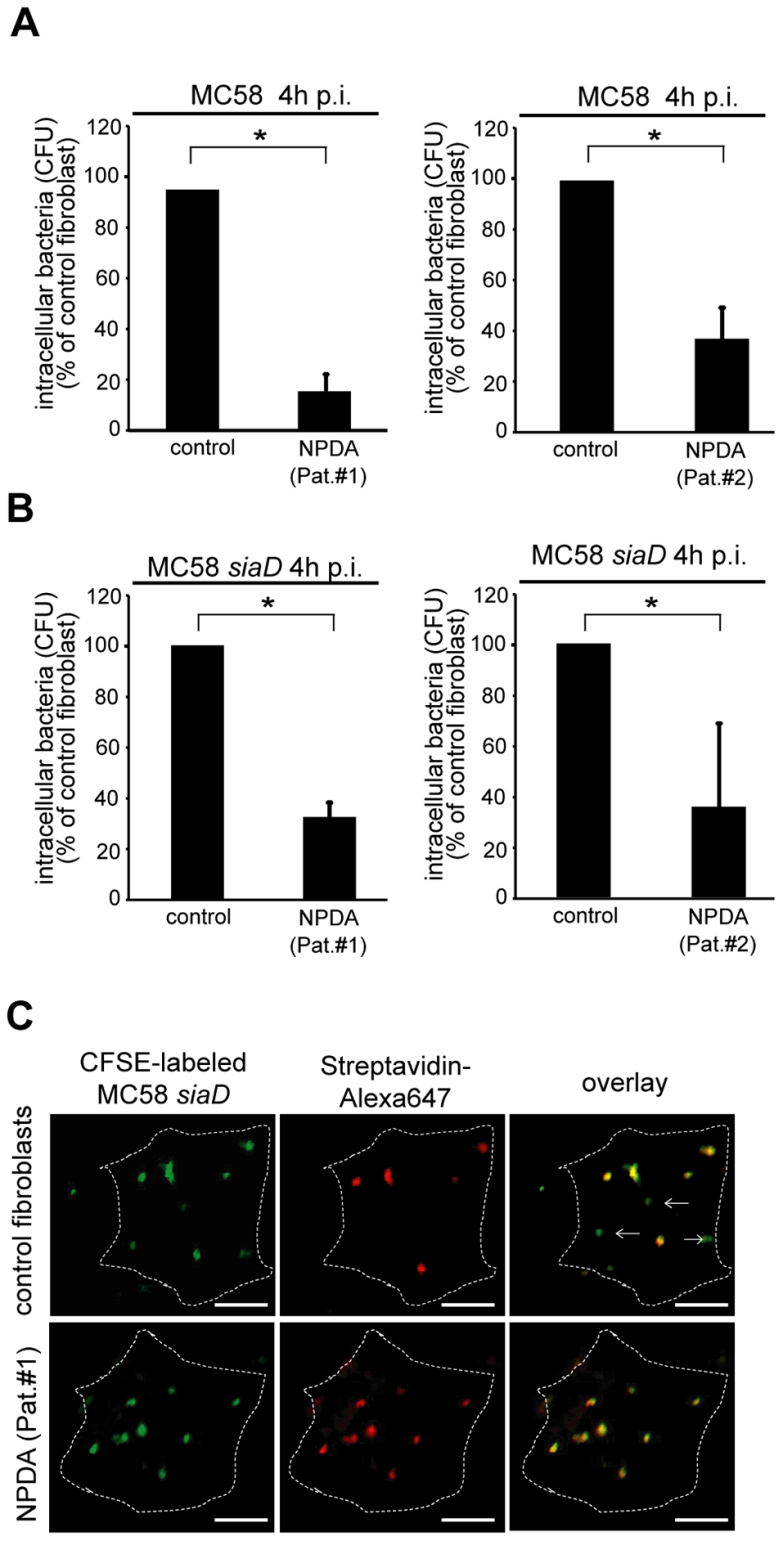
Niemann-Pick disease fibroblasts are significantly less invaded by *N. meningitidis*. (A and B) ASM-deficient fibroblasts of two Niemann-Pick disease type A (NPDA) patients (Pat.#1 and #2) and two healthy control patients were infected with strain MC58 (A) and MC58 *siaD* (B) and number of invasive bacteria were estimated by gentamicin protection assays. The graphs represent mean value ± S.D. of three different independent experiments done in duplicate. * *P*<0.05. (C) Intracellular and extracellular bacteria were detected in infected NPDA (Pat. #1) and healthy controls fibroblasts by microscopy 4 hrs following infection with carboxyfluorescein succinimidyl ester (CFSE)-labeled *N. meningitidis* strain MC58 *siaD*. Extracellular bacteria stain positive with both Streptavidin-Alexa647 (red fluorescence) and CFSE (green fluorescence), whereas intracellular bacteria (arrows) are labeled with CSFE only. Size bars represent 5 µm.

Finally, we comparatively analyzed *N. meningitidis* MC58 and MC58 *siaD* uptake into human fibroblasts generated from healthy donors or patients suffering from Niemann-Pick disease type A (NPDA), a lysosomal storage disease, characterized by a lack of ASM activity. In line with the data obtained for ASM siRNA ablation and in ASM^−/−^ MEFs (Fig. 4D–G), *N. meningitidis* uptake was severely reduced in NPDA-fibroblasts as compared to their wild-type counterparts (Fig. 5A and B). When analyzed by immunofluorescent staining, a significant proportion of cell-associated meningococci localized within fibroblasts of healthy controls, while the frequency of intracellular bacteria was substantially lower in NPDA cells 4 h p.i. ((Fig. 5C and Fig. S5), data shown for MC58 *siaD*) though equivalent amounts of cell-attached bacteria were initially seen in all cultures. Altogether, these findings clearly reveal that ASM activation by meningococci is critical for bacterial uptake, but not adhesion.

### Less invasive MenC isolates induce significantly less ASM activation and ceramide release on HBMEC but not on FaDu cells

**Figure 6:**
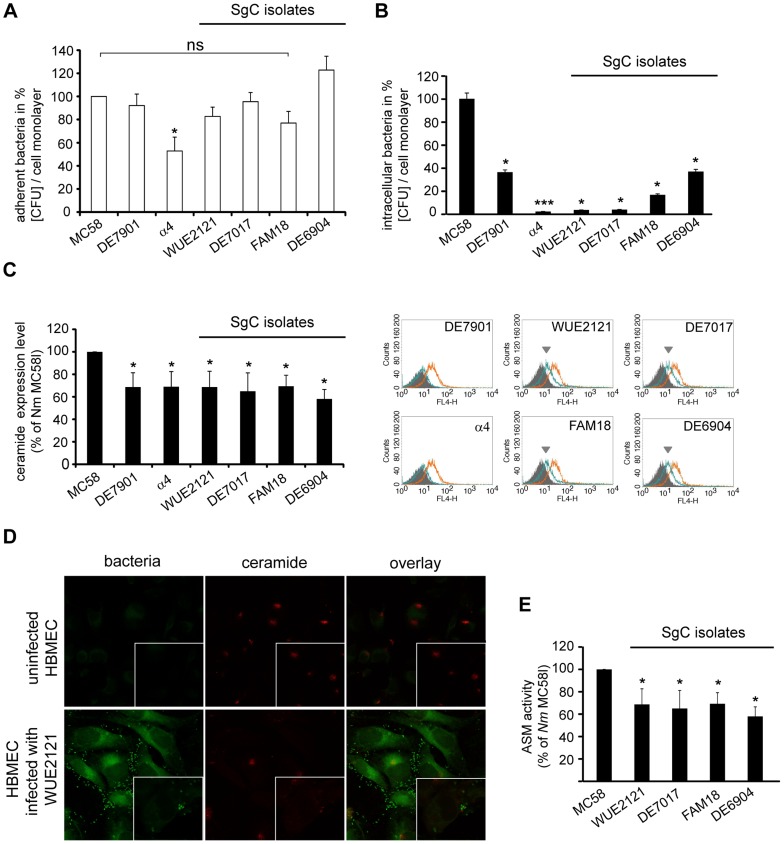
MenC isolates are less efficient at activating ASM and membrane ceramide release on HBMEC. (A) Adherent and (B) intracellular bacteria were evaluated in HBMEC infected with *N. meningitidis* wildtype strain MC58, four MenC strains (WUE2121, DE7017, FAM18, DE6904) and two further MenB strains (DE7901 and α4) for 4 hrs by gentamicin protection assays. The graphs represent mean value ± S.D. of four independent experiments done in duplicate and show the percentage of recovered bacteria compared to MC58. * *P*<0.05. (C) Surface ceramide levels on infected HBMEC were determined by flow cytometry. Representative histograms demonstrating surface expressions of ceramide on HBMEC infected with DE7901, α4, WUE2121, DE7017, FAM18, DE6904 (green lines) compared to *N. meningitidis* strain MC58 (orange lines), uninfected cells served as control (filled grey). (D) Ceramides were detected on HBMEC left uninfected (control) or infected with GFP-expressing strain WUE2121 for 2 hrs by immunofluorescence analysis. (E) Whole cell lysates of cells infected with the four MenC strains and strain MC58 were analyzed for ASM activity. Data (mean ± S.D.) are compiled from three independent experiments performed in triplicate. * *P*<0.05. SgC = serogroup C meningococci.

To analyse whether *N. meningitidis* isolates belonging to different serogroups and/or sequence types might differentially activate ASM and ceramide release, we selected four pathogenic meningococcal isolates of the ST-11/ST-8 cc that belong to serogroup C (MenC strains WUE2121, DE7017, FAM18, DE6904) (Table 1). In gentamicin protection assays, these isolates proved to be significantly less invasive into HBMEC as compared to strain *N. meningitidis* MC58, however, did not differ with regard to adhesion from MC58 (Fig. 6A and B). However, these 4 ST-11 cc isolates proved to be significantly less effective at inducing ceramides in HBMEC than the invasive MC58 strain as determined by flow cytometry (Fig. 6C). Indicating that invasiveness of Neisseria strains links to their ability to cause ASM activation, the activity of the ASM in HBMEC extracts prepared 2 h p.i. with four the MenC strains (WUE2121, DE7017, FAM18, DE6904) was significantly lower than that induced by MC58 (Fig. 6E). These strains, unlike MC58 (Fig. 1), also failed to induce ceramide-enriched membrane platforms (data shown for WUE2121) (Fig. 6D). We extended our study and included two further isolates that belong to serogroup B (strains DE7901 and the carrier isolate α4) (Table 1). Carrier isolate α4 was found to be less adherent to HBMEC than the other strains tested, however, in line with the findings observed for the serogroup C strains, both isolates were found to be significantly less invasive and effective at inducing ceramides on HBMEC (Fig. 6B and C).

**Table 1:** List of *N. meningitidis* strains used in this study.

Strain	Serogroup	Sequence type (ST)	Clonal complex (cc)	Lineage	Source	Country	Year	Ref	Genome
WUE2121	C	11	11	invasive	IMD*	Germany	1997	[63]	–
DE7017		11	11	invasive	IMD	Germany	2000	[64]	–
FAM18		11	11	invasive	IMD	USA	1980	[65]	AM421808
DE6904		8	8	invasive	IMD	Germany	2002	[64]	–
MC58	B	74	32	invasive	IMD	UK	1983	[66]	AE002098
α4	B	19	18	carriage	carrier	Germany	1999	[56]	–
DE7901	B	18	18	carriage	IMD	Germany	2001	[64]	–

IMD* invasive meningococcal disease.

To determine whether ASM activation and ceramide release is specific to brain endothelial cells, we extended our study to epithelial cells and included the human epithelial cell line FaDu. As shown in figure S6, neither adherence nor invasion significantly varied among strains tested at 4 h p.i. Cermide surface levels on FaDu cells generally exceeded those detected on HBMEC, however, did not detectably increase after infection with strain MC58 or four MenC strains as determined by flow cytometry at various time points (Fig. S6C, D).

### Opc is sufficient to induce activation of ASM and ceramide release

**Figure 7:**
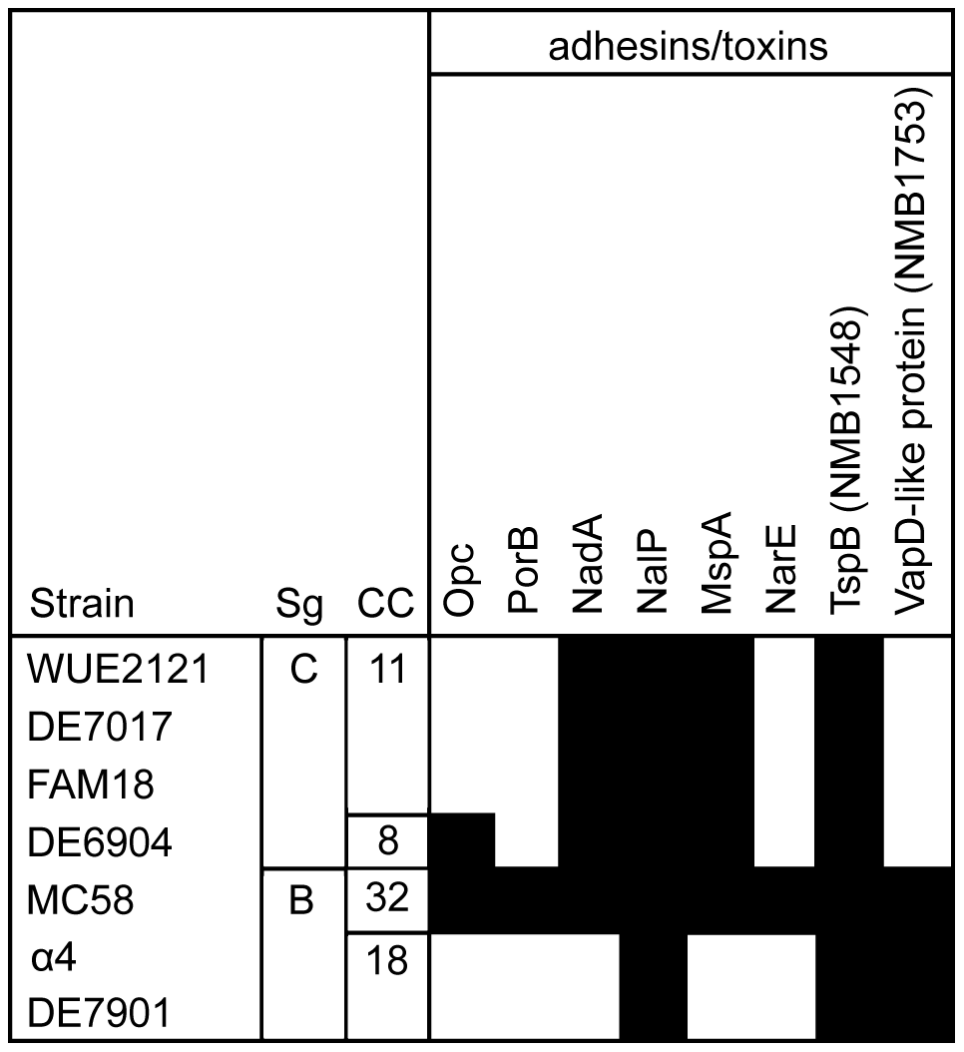
Distribution of surface and virulence-associated proteins. Only surface and virulence-associated proteins are shown that are variably present among the 5 meningococcal strains compared. Sg = serogroup, CC = clonal complex. Figure adapted from [45].

In order to define the meningococcal factor(s) involved in differential ASM activation, we focussed on genes absent from the ST-11 cc strains as compared to *N. meningitidis* MC58. Based on data generated by a previous microarray comparative genome hybridization (mCGH) study [45], 8 genes encoding for surface and virulence-associated proteins were identified as potentially involved in ASM activation also including the Opc outer membrane protein (Fig. 7). This study revealed that the *opc* gene is lacking in the three ST-11 cc strains (WUE2121, DE7017, FAM18), while a hybridization signal was observed for ST-8 cc strain DE6904 in the previous mCGH study, indicating the presence of *opc* in strain DE6904. A detailed PCR amplification of the *opc* gene, however, revealed a deletion of the *opc* gene also in this strain resulting in lack of Opc expression (Fig. S7).

**Figure 8:**
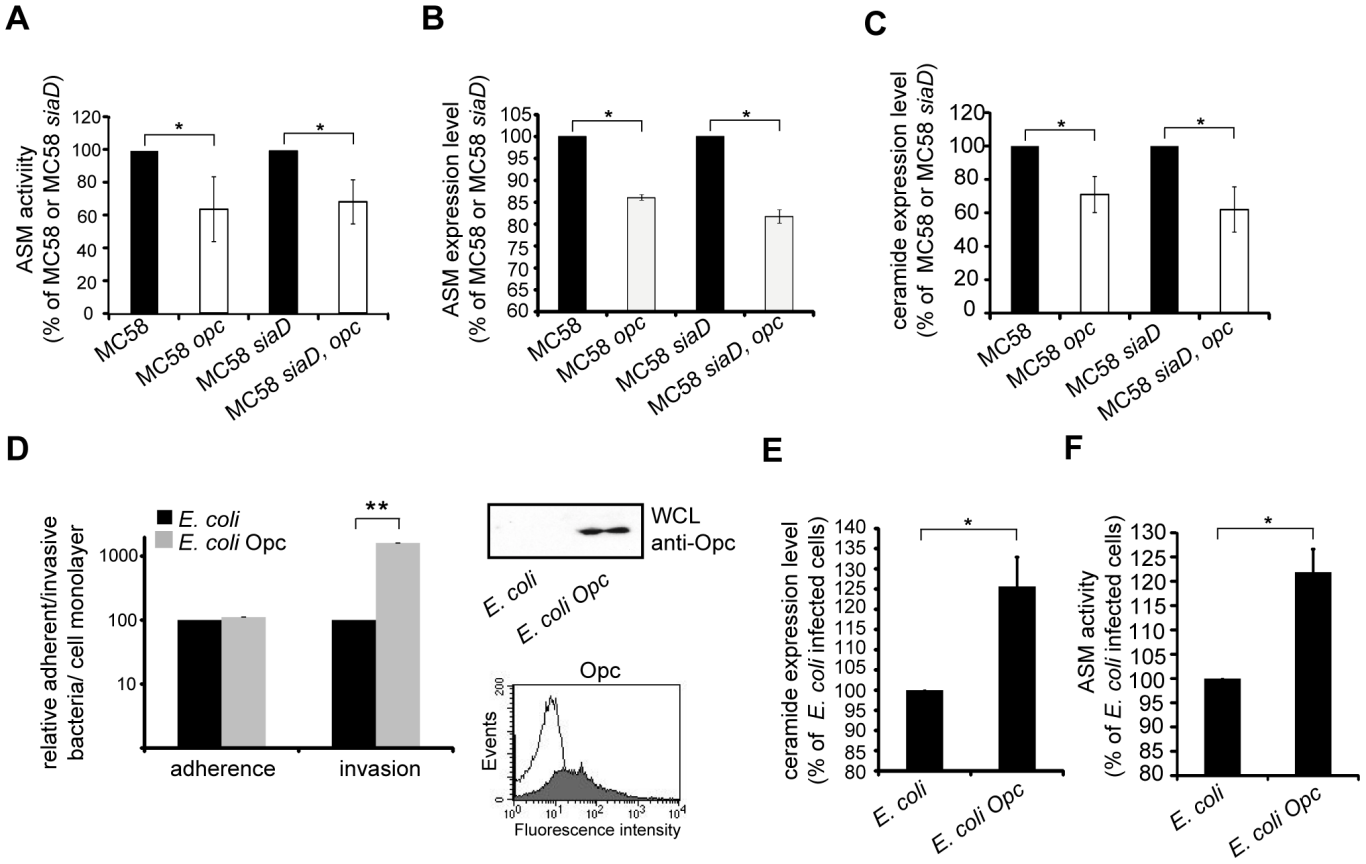
Opc protein contributes to ASM activation and ceramide cell surface release. HBMEC were infected with *N. meningitidis* wildtype strain MC58, isogenic unencapsulated strain MC58 *siaD* and two isogenic strains lacking Opc expression (MC58 *opc* and MC58 *siaD*, *opc*) for 2 h. (A) ASM activity was measured in whole cell lysates of HBMEC infected with *N. meningitidis* wildtype strain MC58 or isogenic mutants. (B) ASM translocation to the outer leaflet was determined by flow cytometry. (C) In parallel, surface ceramide levels on HBMEC were determined in infected cells. All data are mean ± S.D. from three independent experiments performed in triplicate. Values are expressed as the percentage of ceramide expression, ASM translocation and activity in cells infected with Opc-deficient strains compared to parental strains. * *P*<0.05. (D) Expression of recombinant Opc in *E. coli BL21*. Expression of Opc was analyzed either in bacterial lysates by Western Blotting or by flow cytometry analysis using a monoclonal anti-Opc antibody. As a negative control *E. coli* not transformed with recombinant proteins were used. Representative histograms demonstrating surface expressions of Opc on *E. coli* BL21 (filled line) compared to parental strains (open line). Adhesion and invasion to HBMEC of an *E. coli* strain recombinantly expressing Opc (grey bars) or the parent strain (black bars) were determined by gentamicin protection assays. Results represent mean ± S.D. of three independent experiments done in triplicate and show the percentage of recovered bacteria compared to not transformed *E. coli*. ** *P*<0.01. (E and F) Cells were infected with Opc-expressing *E. coli* and parental *E. coli* strain and ceramide accumulation and ASM activity was determined as described above.

To define the role of Opc for ASM activation in detail, we made use of two isogenic Opc-deficient knock out mutants MC58 *opc* and MC58 *siaD*, *opc* [1]. HBMEC were infected with MC58 and isogenic mutants and surface display of ceramide and ASM and total ASM activity were determined. Isogenic Opc-deficient mutants were less efficient at inducing ASM activation (Fig. 8A) and surface display of ASM and ceramide (Fig. 8B and C) compared to wildtype MC58 or isogenic unencapsulated strain MC58 *siaD*. These data indicate that the absence of Opc accounts for differential ASM activation.

To investigate whether the bacterial factor Opc is sufficient in this process, the *opc* gene was cloned under the control of an IPTG inducible prokaryotic expression vector and overexpressed in *E. coli* BL21. Recombinant protein was expressed at high levels in *E. coli* BL21 as demonstrated by immunoblot and flow cytometry analysis (Fig. 8D). We next assessed the properties of the recombinant Opc expressing *E. coli* to adhere to and enter into HBMEC. Therefore, HBMEC were infected for 2 h with *E. coli* BL21 expressing Opc or the parental *E. coli* strain at an MOI of 30. As expected, infection of HBMEC with Opc-expressing bacteria significantly increased internalization (10-fold increase of uptake), but not adherence (Fig. 8D). HBMEC infection with Opc-expressing *E. coli* resulted in a 1.3 fold increase of ceramide release compared to the parental *E. coli* strain (Fig. 8E). Moreover, ASM activity in response to Opc-expressing *E. coli* increased significantly measured at 2 h p.i (Fig. 8F). Together, these data attribute an important role for the Opc protein in enhancement of bacterial uptake driven by ASM activation.

### *N. meningitidis* promotes ErbB2 recruitment in ceramide-enriched platforms

**Figure 9:**
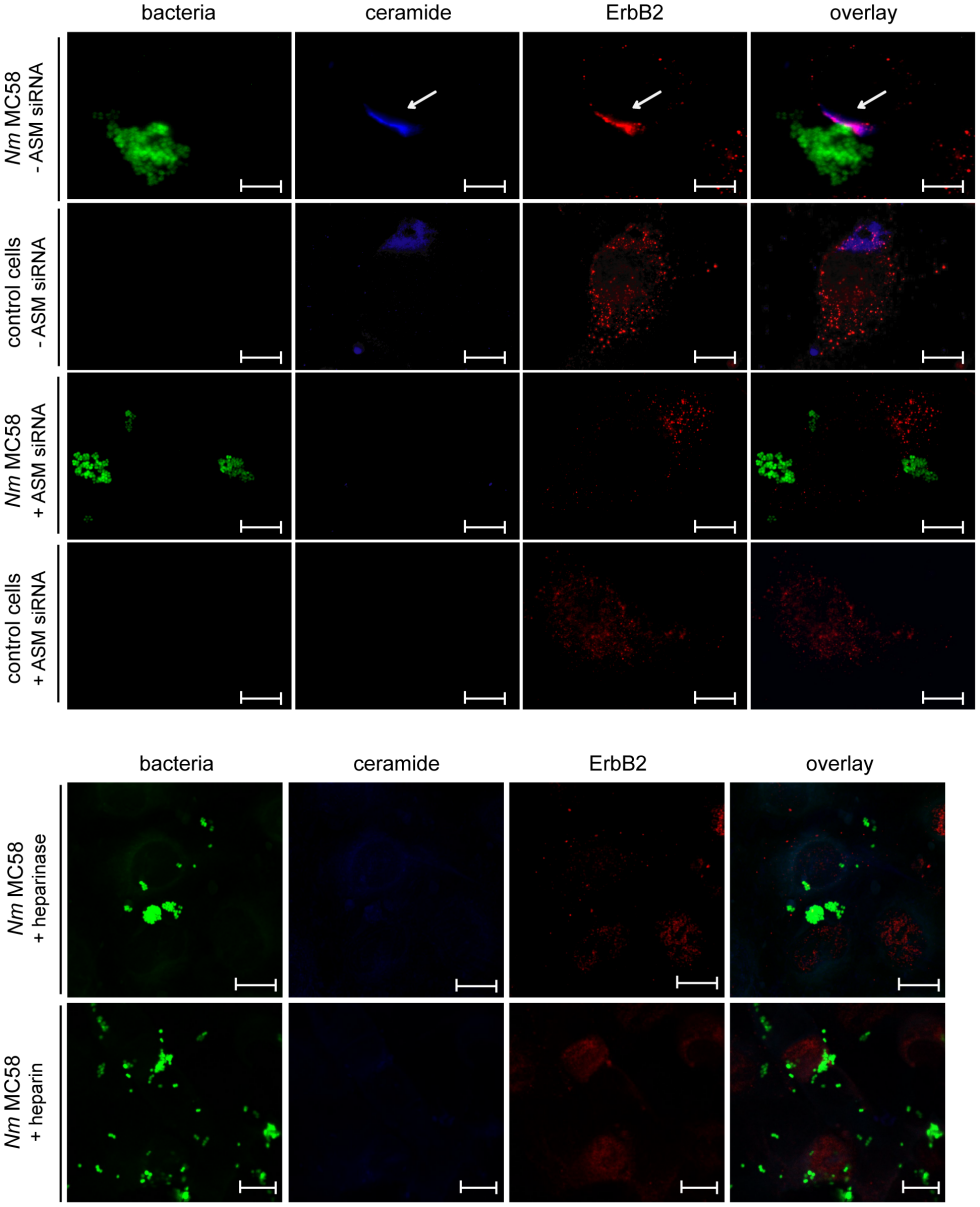
*N. meningitidis* infection promotes ErbB2 recruitment in ceramide-enriched platforms. HBMEC were transfected with siRNAs against ASM (+ ASM siRNA) or control siRNA (− ASM siRNA) for 72 hrs and cells were left uninfected (control cells) (row 2 and 4) or were infected with a GFP expressing isolate of strain MC58 (row 1 and 3). Cells were fixed and stained for ErbB2 with an IgG2a anti-ErbB2 antibody and secondary Cy3-conjugated antibodies, followed by ceramide staining with an anti-ceramide antibody and secondary Alexa350-conjugated anti-mouse-IgM antibody. Infection caused enhanced re-distribution of ErbB2 to the site of attached bacteria. Moreover, confocal microcopy revealed co-clustering of ErbB2 in ceramide-enriched platforms upon infection (arrows). The studies are representative for 3 similar results. Size bars represent 10 µm. (Row 5 and 6) HBMEC were pretreated with 150 mU heparinase or 100 µg/ml heparin and infected with a GFP expressing isolate of strain MC58. Cells were fixed and stained for ErbB2 followed by ceramide staining. The studies are representative for 2 similar results. Size bars represent 10 µm.

There are numerous examples of cellular surface receptors including CD95 [27], CD40 [31] or CD150 [41], which cluster within ceramide-enriched platforms generated in response to ASM activation. We therefore hypothezised that enhancement of *N. meningitidis* uptake into HBMEC by Opc and HSPG driven ASM activation might relate to concentration of receptors involved in meningococcal invasion within these platforms. *N. meningitidis* recruit the tyrosine kinase receptor ErbB2 (Her2/neu) to the sites of bacterial adherence on endothelial cells [46]. Tyrosine phosphorylation of ErbB2 has been shown to be required for efficient uptake by promoting the phosphorylation of the actin-binding protein cortactin [47]. To examine whether this receptor is recruited and trapped into ceramide-enriched membrane platforms, we compared the subcellular distribution of ErbB2 in HBMEC before and after infection with *N. meningitidis*. In uninfected cells, ErbB2 detected in fixed, unpermeablized cells revealed an overall punctuate expression pattern (Fig. 9, second row). Infection with *N. meningitidis* strain MC58 for 2 h, however, caused enhanced re-distribution of ErbB2 to the site where bacteria had attached and formed microcolonies (Fig. 9, first row), where we also detected ceramide-enriched platforms. Formation of ceramide-enriched platforms upon meningococcal exposure was sensitive to ASM knockdown and remarkably, ErbB2 failed to redistribute and cluster under these conditions as well (Fig. 9, third row). To confirm involvement of HSPGs, HBMEC were pretreated with either heparinase (150 mU) or heparin (100 µg/ml) and infected with strain MC58 for 2 h. As shown in figure 9 (rows 5 and 6) formation of ceramide-enriched platforms upon meningococcal exposure was also sensitive to interference with HSPGs and ErbB2 failed to redistribute under these conditions as well. Altogether, these data show that the reorganization of ceramide into large membrane platforms promotes lateral redistribution and clustering of ErbB2, an important receptor involved in *N. meningitidis* uptake.

## Discussion

In this study we provide evidence for an important function of the acid sphingomyelinase (ASM) in Opc-mediated internalization of *N. meningitidis* into brain endothelial cells. Furthermore, we show that activation of ASM occurs downstream of heparan sulfate proteoglycans (HSPGs) and involves phosphatidylcholin phospholipase C (PC-PLC) activity. The importance of ASM activation in *N. meningitidis* uptake was strongly supported by its significant reduction upon experimental (pharmacological and siRNA mediated interference) or natural (NPDA fibroblasts) ablation of the enzyme activity. ASM activation by Opc-expressing *N. meningitidis* caused ceramide release resulting in formation of extended ceramide-enriched membrane platforms, where ASM and, most importantly, the tyrosine kinase receptor ErbB2, involved in the cellular uptake of *N. meningitidis*, are co-displayed.

It is in response to a variety of stimuli also including ligation of certain receptors that lysosomal ASM translocates to the outer leaflet of the cell membrane to catalyze breakdown of sphingomyelin into ceramide. As relevant to our studies, the presence of fatty acids, mono-, di-, and triacylglycerols as well as phosphatidylinositol has been implicated in ASM activation [48], and this especially applies to diacylglycerol (DAG) released in response to by PC-PLC, activated by the related species *N. gonorrhoeae* [37], [43], [44]. Here, we show that interaction of *N. meningitidis* with HSPGs stimulates ASM translocation and activation, and this process also involves PC-PLC activity (Fig. 3). HSPGs function primarily as initial, low affinity co-receptors that act to concentrate pathogens on host cell surfaces, thereby increasing binding to specific secondary receptors. For *N. gonorrhoeae*, ASM activation has been found to be triggered upon Opa-mediated binding to HSPGs [37], whereas ASM activation by *N. gonorrhoeae* in neutrophils involves Opa_52_-mediated binding to CEACAM receptors [38]. CEACAM-mediated activation of ASM by *N. meningitidis* could be excluded in our cell culture model system because this receptor is not expressed on HBMEC (unpublished data). For *N. meningitidis* uptake, it has been established that Opc links the bacterium to vitronectin and/or fibronectin followed by binding to αvβ3 or α5β1-integrins [1], [2]. It is quite possible that the interaction of *N. meningitidis* with integrins stimulates per se or contributes to ASM translocation followed by ceramide release. This can, however, only be addressed by a separate, thorough study which would exceed the scope of the present manuscript.

During the interaction with endothelial cells, pilus mediated adhesion of *N. meningitidis* has been shown to induce the clustering and tyrosine phosphorylation of the host tyrosine kinase receptor ErbB2 [46]. Activation of ErbB2 is required to support *N. meningitidis* internalization [46], by promoting the phosphorylation of the actin-binding protein cortactin and thus it is remarkable that it co-segregates with ASM within ceramide-enriched platforms generated downstream of meningococcal interaction with HSPGs (Figs. 3, 9). This suggests that ASM and ceramide-induced clustering and lateral segregation of ErbB2 in membrane platforms function as an upstream prerequisite for ErbB2-supported meningococcal internalization. For *N. gonorrhoeae*, ASM activation is required for both the entry into professional and non-professional phagocytes [37], [38], however, the formation of large ceramide-enriched membrane platforms and clustering of receptors involved in *N. gonorrhoeae* uptake has not been demonstrated so far.

Our findings are paralleled by the observation that measles virus (MV) interaction with a pattern recognition receptor (DC-SIGN) is followed by SMase catalyzed ceramide release and membrane display of its entry receptor CD150 within ceramide-enriched platforms on dendritic cells [41]. Thus, the generation of ceramide-enriched membrane platforms might be a general motif to mediate uptake of pathogens that applies to a very diverse range of pathogens.

Whereas for MV one receptor is sufficient to promote viral uptake, it is likely that further receptors might be recruited into ceramide-enriched membrane platforms induced upon *N. meningitidis* infection of endothelial cells, since adhesion of *N. meningitidis* to epithelial cells has been shown to induce the formation of a specific molecular complex, referred to as ‘cortical plaque’. ‘Cortical plaques’ are enriched in ezrin, moesin, tyrosine-phosphorylated proteins, ICAM-1, CD44 and EGFR [49], [50]. In addition, meningococcal adhesion on human endothelial cells leads to the redistribution of the Par3/Par6 polarity complex, which is recruited underneath meningococcal microcolonies, weakening the barrier properties of the blood-cerebrospinal fluid barrier [46], [51]. It is tempting to speculate that molecules of the ‘cortical plaque’ or even polarity complex components cluster in ceramide-enriched membrane platforms underneath meningococcal microcolonies.

It is important to note that ASM-inhibition prevented meningococcal uptake, but did not alter adherence regardless of whether the enzyme was inhibited by pharmacological (amitriptyline) or genetic approaches (siRNA ablation, ASM^−/−^ MEFs and NPDA fibroblasts) (Fig. 5). Moreover, there is apparently a peak of ASM translocation at 2 hrs as measured by flow cytometry, which does, however, not resolute subcellular localization of ceramide release. This is much better reflected by detection of ceramide-enriched platforms by immunofluorescence, which, by nature, is not quantitative. Neither ASM alone nor ceramides per se are operative in supporting *N. meningitidis* invasion, but rather act to sort surface and membrane proximal complexes required which may not be completed, but ongoing over the time interval monitored. It is also possible that ceramide release assists in alterations of membrane curvature as required for invasion which may also be time consuming. It has, for instance been shown in an ASM dependent bead uptake assay that increase in membrane order (reflecting ASM activity) peaked already after 5 mins, while bead uptake in macrophages was completed only after 1 hr [52].

Previous studies have shown that *P. aeruginosa* and *Staphylococcus aureus* activate ASM and trigger the formation of ceramide-enriched membrane platforms [39]. However, the bacterial factors responsible for ASM activation have not been identified for these microorganisms so far. In our system, ASM activation and ceramide release were assigned to Opc, and the differential ability of meningococcal clonal complexes to promote ASM activation could be assigned to expression of Opc (Fig. 6 and 8).

Studying the interaction of *N. meningitidis* with host cells is complicated by the high variability of meningococcal isolates which can be classified by multi locus sequence typing (MLST) based on the polymorphisms in seven housekeeping genes [53]. Moreover, meningococcal isolates can be clustered to clonal complexes comprising isolates that share identical nucleotide sequences for at least four MLST loci. Isolates from asymptomatic carriers are more diverse that those recovered from patients with invasive disease [54], [55]. A few so called ‘hyper-invasive’ isolates are responsible for most reported diseases and may spread rapidly through human populations, resulting in countrywide epidemics of meningococcal meningitis [54]–[57]. Two epidemiological studies reported outbreaks of meningococcal strains of the ST-11 cc that tended to cause severe sepsis with fatal outcome but rarely caused meningitis [19], [20]. Employing four MenC isolates belonging to ST-11/ST-8 cc that lack the *opc* gene, we demonstrated that all four MenC strains induced significant less activation and translocation of ASM in endothelial cells, followed by significant less accumulation of ceramide on the cell surface and, therefore, failed to trigger the formation of ceramide-enriched platforms (Fig. 6). In line with a proposed critical role for ASM for meningococcal uptake, all ST-11 cc strains tested in this study proved to be significantly less invasive into HBMEC compared to strain *N. meningitidis* MC58. This indicated a specific stimulation of ASM by the Opc-mediated interaction of *N. meningitidis* with endothelial cells. The contribution of Opc was underlined by using recombinant *E. coli* expressing a functional form of this protein, demonstrating that Opc is sufficient to trigger ASM activation followed by accumulation of ceramide within the outer leaflet of the plasma membrane (Fig. 8). However, since the differences between Opc expressing and non-expressing strains are about 30%, further candidates might also contribute to ASM activation and thus ceramide generation, such as the PorB, NarE or the VapD-like protein, that are also absent in all ST-11/ST-8 cc strains (see Fig. 7).

The critical role of the ASM/ceramide system in uptake of certain bacterial pathogens suggests this system as potential therapeutical target. Amitriptyline used to inhibit ASM in this and several other studies is in clincal use for treatment of depression, and currently tested in a randomized, double-blind, placebo-controlled phase IIb multicenter study for the therapy of patients with cystic fibrosis (CF), where it was found to improve the lung function and to reduce ceramide in the lung cells of CF patients [58]. In contrast to CF, an inherited chronic disease that affects the lungs and digestive system, meningococcal meningitis develops quickly and outcome often depends on how soon antibiotics are given after the illness starts. Approximately 50–60% of patients with systemic meningococcal infection present with clinical symptoms of distinct meningitis. A potentially neuro-protective therapeutic agent blocking the ASM would be administered for a short period, should be well tolerated and, most important, must immediately inhibit the ASM in endothelial cells. Amitriptyline and structurally similar molecules functionally inhibit the ASM by inducing a degradation of the enzyme and, therefore, their action might be too slow. Thus, a drug targeting the ASM in *N. meningitidis* infection should inhibit directly and immediately the ASM to provide a beneficial effect in patients suffering from meningococcal meningitis.

## Materials and Methods

### Bacterial strains

*Neisseria meningitidis* strain MC58, a serogroup B isolate (United Kingdom, 1983) of the ST-32 clonal complex (cc) was characterized as serotype B∶15∶P1.7,16 (kindly provided by E. R. Moxon). Non-encapsulated mutant MC58 *siaD*, Opc-deficient mutant strains MC58 *opc* and MC58 *siaD*, *opc* were previously described [1] (Table 2). Meningococcal isolates of the ST-11/ST-8 cc of serogroup C (*N. meningitidis* strains WUE2121, DE7017, FAM18, DE6904) and isolates of serogroup B (α4 and DE7901) were taken from a collection by Schoen and colleagues [45] and are summarized in table 1. All strains were tested for Opc, NadA and NarE expression (Fig. S7). Polyclonal antibody raised against NarE was kindly provided by Dr. F. Günther (Medical Microbiology and Hygiene, University of Heidelberg). Opc expression was analyzed by PCR using primer pair RA3 5′- CATCTCAAGTCTCGTCATTCC-3′ and RA4: 5′-AGCCTGTGTAAAGATCGATAC-3′, kindly provided by Dr. H. Claus. Lack of *opc* gene was verified by PCR using primer pair RA1 5′- CAAAGCGCACATCACCGTC-3′ and RA2 5′-CCATCAAATGAATATCCATACC-3′. Confocal microscopy was performed with a derivative expressing a green fluorescent protein from the plasmid pEG2-Ery [59], which also contains an erythromycin-resistance gene.

**Table 2:** List of *N. meningitidis* mutant strains used in this study.

Mutant strains	Characteristics
MC58 *siaD*	Isogenic mutant of the pathogenic isolate MC58, deficient in capsule expression, [1]
MC58 *opc*	Isogenic mutant of the pathogenic isolate MC58, deficient in Opc expression, [1]
MC58 *siaD*, *opc*	Isogenic mutant of MC58 *siaD*, deficient in capsule and Opc expression, [1]

### Cell culture and infection assays

The simian virus 40 large T antigen-transformed human brain microvascular endothelial cells (HBMEC) were kindly provided by K. S. Kim [60] and were cultured as previously described [1]. Cells between the 10th and 25th passages were used for infection assays at a density of 1×10^5^ per well with bacteria at a multiplicity of infection (MOI) of 10–30 unless indicated otherwise as described previously [1]. Infections were carried out in the presence of 10% human serum (HS) supplemented RPMI medium. HS were derived from a serum pool (voluntary staff) and heat-inactivated for 30 min at 56°C [1]. Wildtype strain *N. meningitidis* MC58 and the isogenic capsule deficient mutant were tested repeatedly for pili, Opa and Opc expression before application to infection assays and after reisolation from the cell culture using Western blot analysis. HBMEC/ciβ was kindly provided by Tomomi Furihata [61]. HBMEC/ciβ were generated from a human primary BMEC-derived cell line immortalized with both the temperature-sensitive mutant of the simian virus 40 large tumor antigen (tsSV40T) and the human telomerase reverse transcriptase subunit (hTERT) [61]. Human ASM-deficient fibroblasts were obtained from patients with Niemann-Pick Disease type A (NPDA) and maintained in RPMI medium 1640. Mouse embryonic fibroblasts (MEFs) were derived from ASM knockout mice or wildtype embryos and grown in Dulbecco's modified Eagle's medium (DMEM) supplemented with 10% fetal calf serum (FCS). FaDu (ATCC HTB 43), an epithelial-like human pharynx cell line from cell carcinoma, was maintained in DMEM supplemented with 10% FCS.

### Reagents and antibodies

When indicated, cells were exposed to amitriptyline (5 µM, 7.5 µM and 10 µM), Heparinase III (from Flavobacterium heparinum [H8891]; 75 mU and 150 mU) and heparin (heparin sodium salt; 10 µg/ml, 50 µg/ml and 100 µg/ml) (all: Sigma-Aldrich (Sigma-Aldrich, Taufkirchen, Germany)), phosphatidyl choline-specific phospholipase C (PC-PLC) inhibitor [D609] (100 µM) or phospholipase A_2_ inhibitor AACOCF_3_ (0.1 µM, 5 µM and 10 µM) or phospholipase A_2_ inhibitor FIPI (20 nM, 100 nM and 750 nM) (all: Tocris Bioscience, Bristol, UK). 6-Hexadecanoylamino-4-methylumbelliferyl-phosphorylcholine (HMU-PC) was purchased from Moscerdam Substrates (Amsterdam, The Netherlands). D609, AACOCF_3_, FIPI and amitriptyline did not affect the viability of bacteria or HBMEC judged by survival assays and microscopic or flow cytometry examination. For flow cytometry, fluorescence microscopy and Western blotting analyses the following primary antibodies were used: mouse monoclonal antibody (mAb) anti-ASM IgG (clone ab74281, Abcam, Cambridge, UK), mouse mAb anti-ceramide IgM (clone MID 15B4, Alexis), polyclonal rabbit anti-ASM IgG (H-181, Santa Cruz), mouse mAb anti-ErbB2/Her2/neu IgG2a (clone Neu (C3), Santa Cruz). Secondary Cy3-conjugated goat anti-mouse IgM, goat anti-rabbit IgG (H+L) Cy3 and Cy5 conjugates, Cy5-conjugated goat anti-mouse IgM, Fluorescein isothiocyanate (FITC)-conjugated goat anti-rabbit IgG (H+L) and Tetramethylrhodamine isothiocyanate (TRITC)-conjugated goat anti-rabbit IgG (H+L) (all: Dianova (Dianova, Hamburg, Germany)). Alexa350-conjugated goat anti-mouse IgM (μ-chain specific) was from Invitrogen. Nitrocellulose membranes were incubated with horseradish peroxidase (HRP)-conjugated secondary goat anti-mouse IgG (bacteria) (Sigma-Aldrich, St.Louis, MO) or HRP-conjugated secondary goat-anti-mouse IgM (spot assay) (Dianova).

### Expression of meningococcal proteins in *E. coli*

In order to generate a Opc-expressing *Escherichia coli* strain, the *opc* gene was amplified by PCR from chromosomal DNA of *N. meningitidis* strain MC58 using the primer pair OpcA-MC58-sense (5′-CGGATCCATGGGCAAAAAAACAGTTTTT -3′, *NcoI* site) and OpcA-MC58-antisense (5′- CCCGCTCGAGTCAGAATTTTATGCCGACGCG -3′, *XhoI* site). The PCR product was digested with *NcoI*/*XhoI* and cloned in pET28a(+) (Novagen). The pET28a(+) vector encoding Opc protein were transformed in *E. coli* BL21 (DE3; from Novagen). Cloned *opc* gene was verified by sequence analysis. Opc expression was controlled by immunoblot analysis using monoclonal anti-Opc antibodies (clone B306, kindly provided by M. Achtman).

### Western blot

For Western blot experiments, overnight cultures of meningococci were inoculated into Polyvitex (1% (v/v)) supplemented Proteose Peptone medium (PPM+) and grown to mid-log phase (OD_600_ = 0.5–0.6) at 200 r.p.m and 37°C. 4×10^8^ bacteria were harvested by centrifugation, resuspended in 50 ml of sample solution (25% Tris 250 mM pH 6.8, 50% Glycerin, 10% SDS (w/v) and 25% β-Mercaptoethanol) and heated to 100°C for 10 min. Protein concentration of each sample was measured using the BCA assay (Thermo Scientific, USA) and 10 µl of a 300 mg/ml final concentration of denatured protein per sample was used for electrophoresis, blotting and incubation with anti-Opc, anti-NadA or anti-NarE. Detection was performed with the Pierce chemiluminescence Western blotting kit (Thermo Scientific, USA).

### Ceramide detection

Cell surface ceramide was detected using a spot assay previously described [41]. In brief, 5×10^5^ cells were fixed in 3.7% formaldehyde for 15 min at 37°C, scraped from the culture flask, pelleted, washed twice with 1× PBS and incubated with anti-ceramide IgM (clone MID 15B4) for 45 min at RT. Cell bound antibody was desorbed for 30 sec in 20 µl 100 mM glycine-HCl, pH 3.0, and spotted on nitrocellulose following neutralization in 20 µl Tri-HCl, pH 8.0. Antibody was detected with secondary peroxidase -conjugated goat-anti-mouse IgM (1∶2500) and after washing (3 times for 10 min in PBS-Tween (PBS-T)), enhanced chemiluminescence was used to visualize spots.

### ASM activity assay

ASM activity assays were performed using a commercial assay kit according to the manufacturer's instructions (Sphingomyelinase fluorometric assay Kit, Cayman Chemical, Ann Arbor, MI). In brief, 5×10^5^ cells/well were seeded on gelatin-coated 6-well tissue culture plates (Sarstedt Inc., Newton, NC) and infected 72 hrs later at an MOI of 10. Cells were harvested after removal of the medium and a washing step in PBS by scraping from the culture flask in 500 µl ice cold PBS and pelleted by centrifugation at 800 g for 10 min at 4°C. Pellets were resuspended in 300 µl ice cold SMase acid solution, briefly sonicated (20 pulses, 1 s), and lysates were separated from cellular debris by centrifugation at 20,000× g for 10 min at 4°C. Pellets were resolved in 100 µl SMase acid dilution buffer. For the reaction 10 µl sample per well was applied and incubated for 30 min with Sphingomyelin substrate at 37°C following 100 µl enzyme mixture (as provided by the manufacturer) for 30 min at 37°C. The subsequent analysis was performed by using an Infinite F200 Pro Reader (Tecan Group, Maennedorf, Switzerland) with an excitation wavelength of 540 nm and an emission wavelength of 590 nm.

### NSM activity assay

NSM activity assays were performed using the fluorescent substrate 6-Hexadecanoylamino-4-methylumbelliferyl-phosphorylcholine (HMU-PC) (Moscerdam). In brief, 5×10^4^ cells/well were seeded 72 h prior to infection on gelatin-coated 24-well tissue culture plates (Sarstedt Inc., Newton, NC), grown to a density of approximately 2×10^5^ cells per well and infected at an MOI of 10. Cells were trypsinised in 200 µl trypsin following addition of 800 µl RPMI medium transferred to a new 1.5 ml tube. The suspensions were centrifuged at 800 g for 5 min and pellets were resuspended in 100 µl lysis buffer (20 mM Hepes pH 7.4, 2 mM EDTA pH 8.0, 5 mM EGTA pH 8.0, 1 mM sodium vanadate, 10 mM beta-glycerolphosphate, 5 mM DTT, protease inhibitor cocktail). After 5 cycles of freezing-thawing in methanol/dry ice, cell suspensions were centrifuged for 5 min at 1,600 rpm at 4°C. Supernatants were transferred to a 1.5 ml glass tube, overfilled with 500 µl PBS and pelleted for 1.5 h at 26,000 rpm at 4°C in an SW28 rotor (Beckman). The pellets containing the membrane fractions were resuspended in 40 µl lysis buffer and vortexed gently. Finally, 10 µl resuspension buffer (20 mM Hepes pH 7.4, 2 mM EDTA pH 8.0, 10 mM MgCl_2_, 0.1 mM sodium vanadate, 10 mM beta-glycerolphosphate, 5 mM DTT, 7,5 mM ATP and 0.2% Triton-X-100), 10 µl HMU-PC substrate, 10 µl membrane fraction and 1.8 µl Tric HCl (pH 8.0) were mixed and incubated at 37°C overnight. Reaction was stopped by addition of 200 µl stop buffer and enzyme reaction was measured on an Infinite F200 Pro Reader with an excitation wavelength of 404 nm and an emission wavelength of 460 nm.

### Assay of PC-PLC activity

PC-PLC activity assays were performed using a commercial assay kit according to the manufacturer's instructions (EnzChek, Invitrogen). Cell sonicates were prepared according to the methods described by Gomez-Cambronero *et al.* [62]. For the reaction 100 µl sample (in 1X PC-PLC buffer) per well was applied and incubated with 100 µl of 1X PC-PLC substrate (dye-labelled glycerol-phosphoethanolamine) (as provided by the manufacturer) for 30 min at RT. The cleavage releases the dye-labeled diacylglycerol, which produces a positive fluorescence signal that can be measured. The subsequent analysis was performed by using an Infinite F200 Pro Reader with an excitation wavelength of 490 nm and an emission wavelength of 520 nm.

### siRNA transfection

An ASM targeting siRNA were designed to generate dsRNA for post- transcriptional gene silencing of the ASM gene. The following sequences were used for siRNA knockdown experiments: ASM sense 5′-GGUUACAUCGCAUAGUGCCTT-3′ and ASM antisense 5′-GGCACUAUGCGAUGUAACCTT-3′. SiRNA oligonucleotides were obtained from Sigma-Aldrich (Sigma-Aldrich, St.Louis, MO). As negative control scrambled non-targeted control siRNA [sc-37007] (Santa Cruz Biotechnology, Santa Cruz, CA) were used. Control nonsilencing siRNA and ASM siRNA were transiently transfected into HBMEC growing in HBMEC medium using 3 µl of HiPerfect Transfection Reagent (Qiagen) according to the manufacturer's instructions. Protein knockdown efficiencies by siRNA transfection were verified by reverse transcription PCR after 72 h of transfection.

### Reverse transcription PCR

RNA was extracted from HBMEC tissue using RNeasy Mini kits (Qiagen). cDNA was synthesized from 1.0 µg of RNA using Oligo-dT primers (Invitrogen) using Superscript II Reverse Transcriptase (Invitrogen) according to the manufacturer's instructions. For PCR, the mixture was denaturated at 95°C for 3 min and the target genes were amplified by 35 cycles of reaction: ASM (95°C 30 s, 61°C 30 s, 72°C 1 min) or β-actin (95°C 30 s, 61°C 30 s, 72°C 1 min). Primers used for real time PCR are as follows: forward human ASM, 5′-CCTTTTGATATGGTGTACTTGGAC-3′, reverse human ASM, 5′-GTAATAATTCCAGCTCCAGCTCT-3′; forward human β-actin, 5′-GGACTTCGAGCAAGAGATGG-3′, reverse human β-actin, 5′-AGCACTGTGTTGGCGTACAG-3′.

### Flow cytometry

HBMEC were washed twice with PBS and resuspended in ice-cold fluorescence-activated cell sorting (FACS) buffer (5% FCS and 0.1% NaN3 in PBS). 4×10^5^ cells were incubated for 90 min with either mouse mAb anti-ASM IgG antibody (1∶250 in FACS buffer) or mouse mAb anti-Ceramide IgM antibody (mAb 15B4) (1∶30 in FACS buffer), followed by washing and incubation with Cy5-conjugated goat anti-mouse IgM (1∶300 in FACS buffer) or Cy5-conjugated goat anti-mouse IgG. After the incubation cells were washed and resuspended in 500 µl FACS buffer for the measurement. 10,000 cells were analyzed using a FACSCalibur (BD Bioscience) and the CellQuest Pro software (version 5.2). For determination of Opc expression on the surface of *E. coli*, *E. coli* BL21(DE3) pET-*opc* cells were grown and expression of the Opc protein was induced by adding IPTG to a final concentration of 1 mM and incubating the cells for another hour at 37°C under shaking (200 rpm). Cells were harvested by centrifugation and washed twice with PBS suspended to a final OD_600_ of 0.1/ml for further experiments. Cells were again centrifuged and resuspended in 1 ml FACS buffer. After centrifuging the cells for 10 min at 4,500 g (VWR International GmbH, Darmstadt, Germany), the obtained cell pellet was suspended with 200 µL of mouse anti Opc antibody (clone B306, diluted 1∶100 in FACS buffer) and incubated for 60 min on ice. Subsequently cells were washed twice with FACS buffer and bacterial pellets were resuspended in 20 µL of secondary Cy5-conjugated anti-mouse IgG antibody (1∶300 in FACS buffer) and incubated for 30 min in the dark on ice. After washing twice, the cell pellets were finally suspended in 1.5 mL of FACS buffer. The samples were analyzed using a flow cytometer using a FACSCalibur and the CellQuest Pro software (version 5.2).

### Fluorescence microscopy

#### Ceramide staining

Cells grown on glass coverslips to confluence were infected with GFP-expressing *N. meningitidis* strain MC58 (MOI of 30) for indicated time points, washed, fixed for 15 min in 3.7% paraformaldehyde in PBS, incubated for 45 min in blocking buffer (PBS/1% FCS/2% BSA) and stained with primary anti-ceramide IgM antibody mAb 15B4 (1∶50 in blocking buffer) for 45 min, followed by three PBS washing steps and incubation with Cy3-labeled anti-mouse IgM antibodies (1∶200 in blocking buffer) for further 45 min. Stained cells were viewed and photographed by using a Leica SP5 (Leica Microsystems, Wetzlar, Germany). Images were processed with LAS AF (Leica) and ImageJ software and documented using Adobe Photoshop CS. *MenC strain and ceramide:* Cells were infected with GFP expressing *N. meningitidis* strain WUE2121 (MOI of 30), a derivate of WUE2121 expressing GFP from the plasmid pEG2-Ery [59] as described for strain MC58.

#### Niemann-Pick disease type A cell staining

For determination of extra- and intracellular bacteria a double cycle antibody staining protocol was used, as recently described [1]. Samples were viewed and photographed by using a Zeiss Axio Imager.Z1 fluorescence microscope.

#### Co-localisation of ASM and ceramide

For co-localisation of ASM and ceramide, cells were infected with bacteria for indicated time points. After the indicated time of stimulation, the cells were washed and fixed for 15 min in 3.7% paraformaldehyde in PBS. Cells were washed, blocked for 45 min and incubated for 45 min each with a rabbit anti-ASM antiserum (1∶100 dilution, clone H-181, Santa Cruz) and anti-ceramide mAb 15B4 (1∶100 dilution), respectively. The anti-ASM antibody was visualized with a Cy5-conjugated anti-rabbit Ig antibody and the anti-ceramide antibody was visualized with a Cy3-conjugated anti-mouse IgM antibody. Co-localization was monitored with a Leica SP5 confocal laser scanning microscope using a Plan Apo 63×/1.4 NA oil immersion objective. Fluorescence signals of labeled specimens were serially recorded with appropriate excitation wavelengths and emission bands for Cy3 or Cy5, respectively, to avoid bleedthrough. Images were processed as described above.

#### Co-localisation of ErbB2 and ceramide

For co-localisation of ErbB2 and ceramide, cells were stained with primary anti-ErbB2 IgG2a (1∶ 150 in blocking buffer) and anti-ceramide antibody as described above. The anti-ErbB2 antibody was visualized with a Cy3-conjugated anti-rabbit Ig antibody, whereas the anti-ceramide antibody was visualized with an anti-mouse (μ-chain specific) Alexa350-conjugated secondary antibody.

### Statistical analysis

Statistical differences between groups were calculated using the Student's unpaired *t*-test (two-tailed) using Excel. *P*-values ≤0.05 were considered significant, *P*-values ≤0.01 were considered highly significant.

## Supporting Information

Figure S1 ***N. meningitidis*** **induces a transient formation of ceramide-enriched membrane platforms on brain endothelial cells (HBMEC).** (A) Lower magnification image of HBMEC that were infected with a GFP-expressing wildtype strain MC58 for 2 h (bottom panels) or left uninfected (control cells, upper panels), fixed, left intact, stained with anti-ceramide antibodies and secondary Cy3-conjugated anti-mouse-IgM antibodies and analyzed by confocal microscopy. The data are representative for 3 similar studies. Size bars represent 20 µm. (B) HBMEC were infected with wildtype strain MC58 for a 4 hrs period, fixed after 1 h, 2 h, 3 h and 4 h p.i., left intact, stained with anti-ceramide antibodies and secondary Cy3-conjugated anti-mouse-IgM antibodies The data are representative for 2 similar studies. Size bars represent 20 µm.
(TIF)

Figure S2 ***N. meningitidis*** **infection does not activate the neutral sphingomyelinase (NSM) in HBMEC.** HBMEC were infected with *N. meningitidis* wildtype strain MC58 for the indicated time points or left uninfected (control). Membrane fractions of infected and uninfected control cells were prepared and analyzed for NSM activity as described under “Materials and Methods”. The data are the mean ± S.D. from three independent experiments performed in triplicate. ns = not significant.
(TIF)

Figure S3 ***N. meningitidis*** **infection causes membrane ceramide accumulation on HBMEC/ciβ.** Surface ceramide release on *N. meningitidis* MC58 infected HBMEC/ciβ (for control, uninfected HBMEC/ciβ) was determined by flow cytometry. An isotype antibody served as a negative control in staining experiments. All data show mean values ± S.D. of three independent experiments done in duplicate. * *P*<0.05, ** *P*<0.01, *** *P*<0.001, relative to uninfected control cells.
(TIF)

Figure S4 ***N. meningitidis*** **infection activates phosphatidylcholine-specific phospholipase C (PC-PLC) in HBMEC.** HBMEC were treated with the PC-PLC inhibitor D609 (100 µM) 30 min prior to infection with *N. meningitidis* MC58 (open bars) or were left untreated (black bars), and PC-PLC activity was determined at indicated time points using a commercial assay kit. Non-infected cells served as control. Results represent mean ± S.D. of two independent experiments done in triplicates. * *P*<0.05.
(TIF)

Figure S5 **Immunofluorescence of Niemann-Pick disease (NPDA) fibroblasts infected with** ***N. meningitidis*** **.** Lower magnification image of NPDA (Pat. #1) and healthy controls fibroblasts infected with CFSE-labeled *N. meningitidis* strain MC58 *siaD at* 4 hrs p.i. Extracellular bacteria stain positive with both Streptavidin-Alexa647 (red fluorescence) and CFSE (green fluorescence), whereas intracellular bacteria (arrows) are labeled with CSFE only.
(TIF)

Figure S6 **Estimation of adherence and invasion of** ***N. meningitidis*** **strains to FaDu cells and release of ceramide.** (A) FaDu cells were infected with strain MC58 and four MenC strains (WUE2121, DE7017, FAM18, DE6904) for 4 hrs p. i. and the number of adherent and invasive bacteria was estimated. The graphs represent mean value ± S.D. of three different independent experiments done in duplicate. * *P*<0.05. (B) Relative amount of intracellular bacteria compared to strain MC58. (C) FaDu cells were infected with strain MC58 for a 4 hrs time period and surface ceramide release was determined at indicated time points by flow cytometry. Representative FACS analyses on FaDu cells infected with strain MC58, control, uninfected FaDu. Open histograms indicate isotype control, filled histograms show ceramide staining. (D) Surface ceramide release on FaDu cells that were either left uninfected (control) or were infected with MC58 and four MenC strains (WUE2121, DE7017, FAM18, DE6904) was determined by flow cytometry. Open histograms indicate isotype control, filled histograms show ceramide staining. All data show mean values ± S.D. of three independent experiments done in duplicate. ns = not significant.
(TIF)

Figure S7 **Characterization of expression of adhesins of MenC strains used in this study.** Western blot analysis of Opc, Opa, NadA and NarE expression of whole bacterial lysates: MC58, DE6894, WUE2121, DE7017, FAM18, DE6904. As a negative control appropriate isogenic knock out mutants were included: MC58 *opc*, MC58 Opa-, MC58 *nadA* and MC58 *narE*.
(TIF)
