# Supplementary figures and images for: Differential Activation of Acid Sphingomyelinase and Ceramide Release Determines Invasiveness of Neisseria meningitidis into Brain Endothelial Cells
Source: PLoS Pathog. 2014 Jun 12;10(6):e1004160. doi: 10.1371/journal.ppat.1004160 (PMC4055770; doi:10.1371/journal.ppat.1004160)

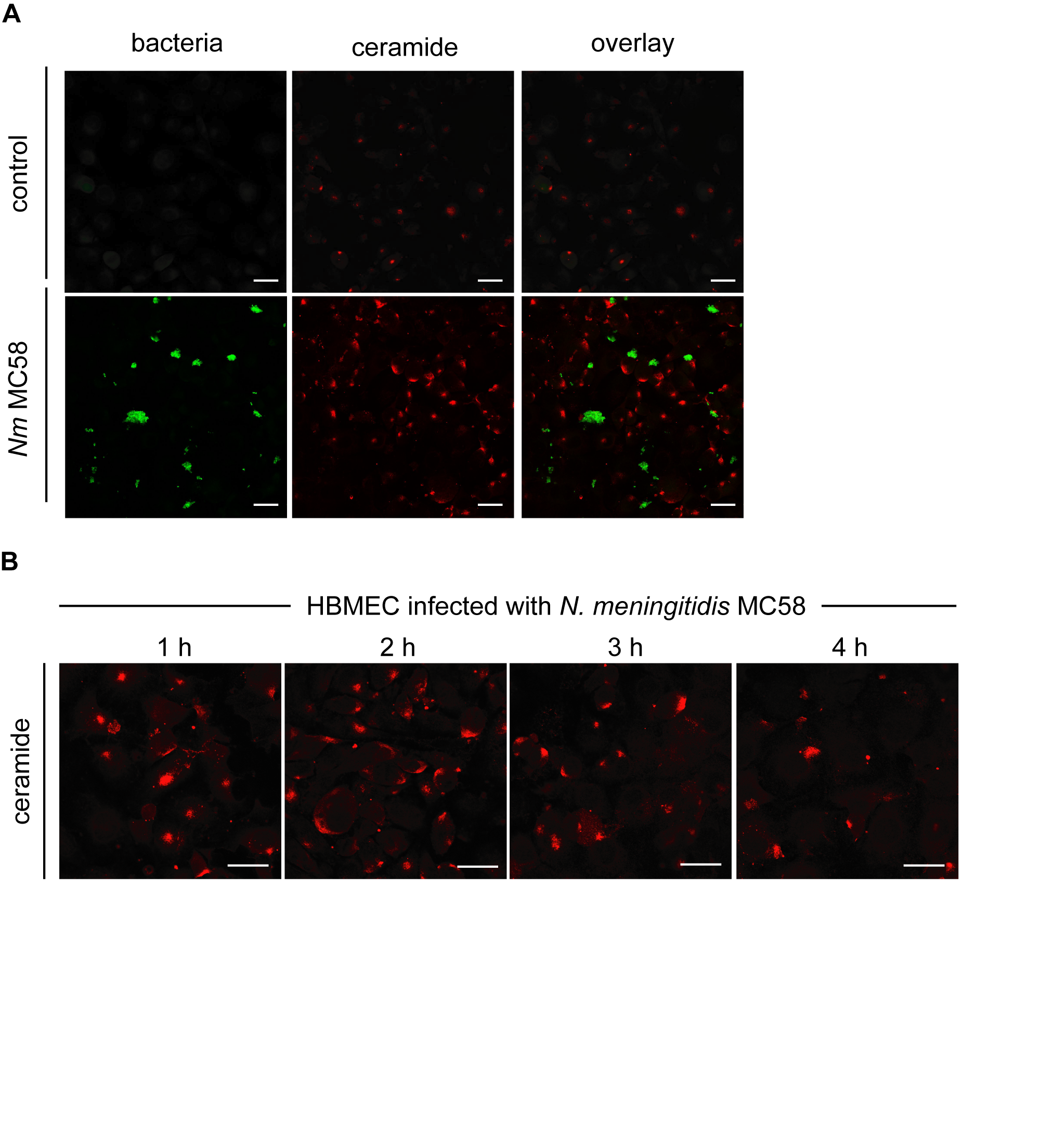

Supplement: Figure S1 — N. meningitidis induces a transient formation of ceramide-enriched membrane platforms on brain endothelial cells (HBMEC). (A) Lower magnification image of HBMEC that were infected with a GFP-expressing wildtype strain MC58 for 2 h (bottom panels) or left uninfected (control cells, upper panels), fixed, left intact, stained with anti-ceramide antibodies and secondary Cy3-conjugated anti-mouse-IgM antibodies and analyzed by confocal microscopy. The data are representative for 3 similar studies. Size bars represent 20 µm. (B) HBMEC were infected with wildtype strain MC58 for a 4 hrs period, fixed after 1 h, 2 h, 3 h and 4 h p.i., left intact, stained with anti-ceramide antibodies and secondary Cy3-conjugated anti-mouse-IgM antibodies The data are representative for 2 similar studies. Size bars represent 20 µm. (TIF) [file ppat.1004160.s001.tif]

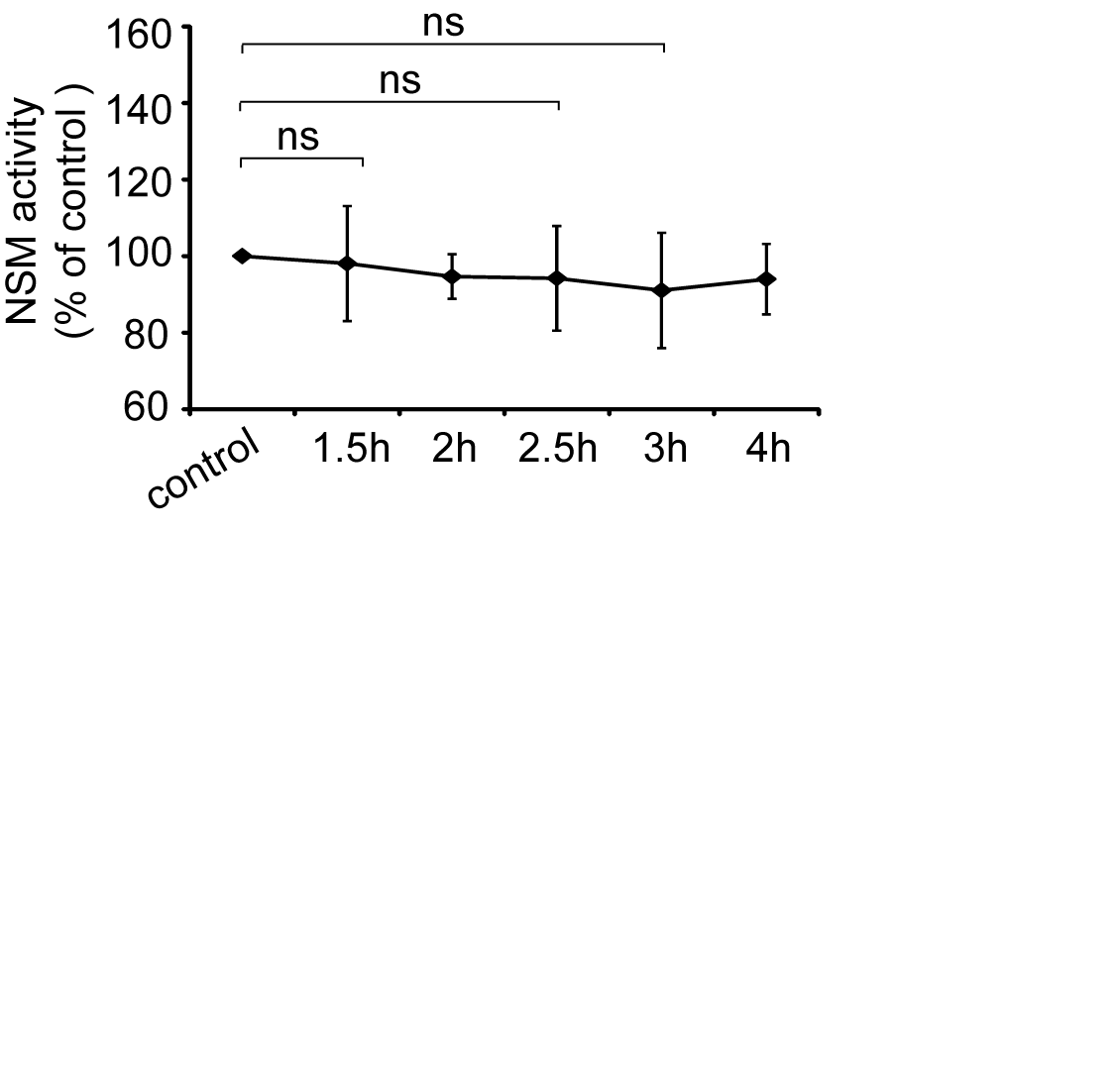

Supplement: Figure S2 — N. meningitidis infection does not activate the neutral sphingomyelinase (NSM) in HBMEC. HBMEC were infected with N. meningitidis wildtype strain MC58 for the indicated time points or left uninfected (control). Membrane fractions of infected and uninfected control cells were prepared and analyzed for NSM activity as described under “Materials and Methods”. The data are the mean ± S.D. from three independent experiments performed in triplicate. ns = not significant. (TIF) [file ppat.1004160.s002.tif]

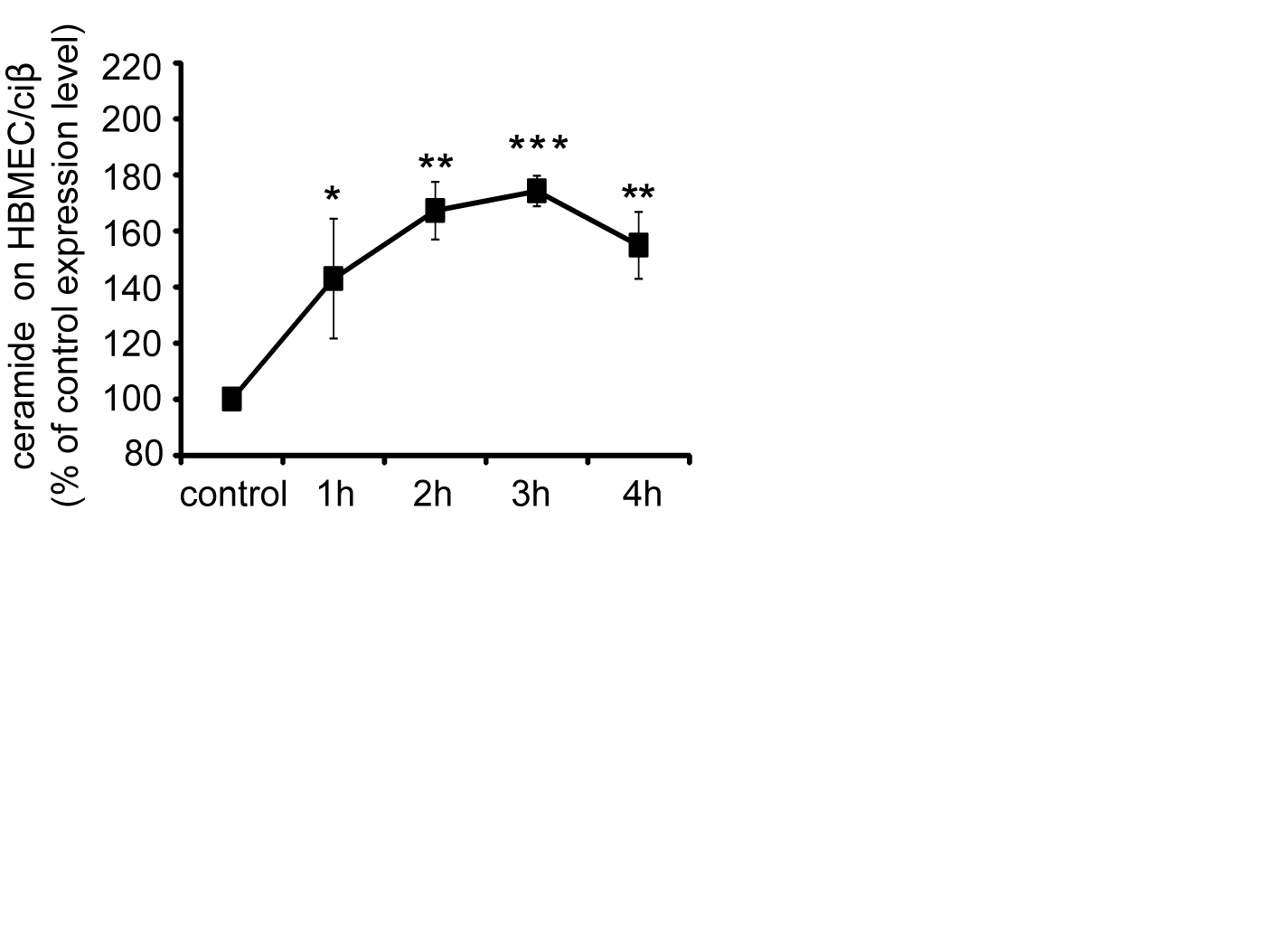

Supplement: Figure S3 — N. meningitidis infection causes membrane ceramide accumulation on HBMEC/ciβ. Surface ceramide release on N. meningitidis MC58 infected HBMEC/ciβ (for control, uninfected HBMEC/ciβ) was determined by flow cytometry. An isotype antibody served as a negative control in staining experiments. All data show mean values ± S.D. of three independent experiments done in duplicate. * P<0.05, ** P<0.01, *** P<0.001, relative to uninfected control cells. (TIF) [file ppat.1004160.s003.tif]

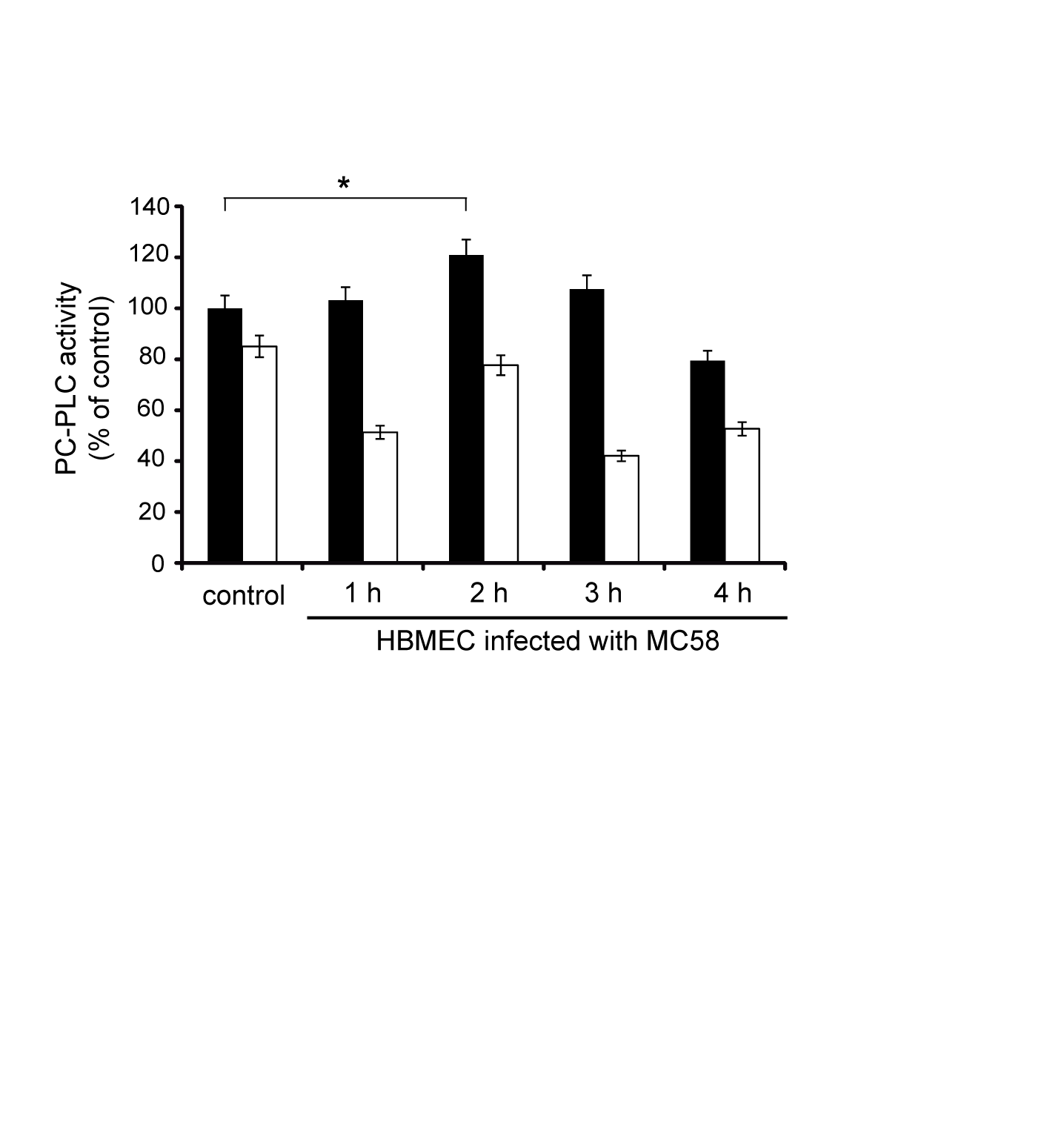

Supplement: Figure S4 — N. meningitidis infection activates phosphatidylcholine-specific phospholipase C (PC-PLC) in HBMEC. HBMEC were treated with the PC-PLC inhibitor D609 (100 µM) 30 min prior to infection with N. meningitidis MC58 (open bars) or were left untreated (black bars), and PC-PLC activity was determined at indicated time points using a commercial assay kit. Non-infected cells served as control. Results represent mean ± S.D. of two independent experiments done in triplicates. * P<0.05. (TIF) [file ppat.1004160.s004.tif]

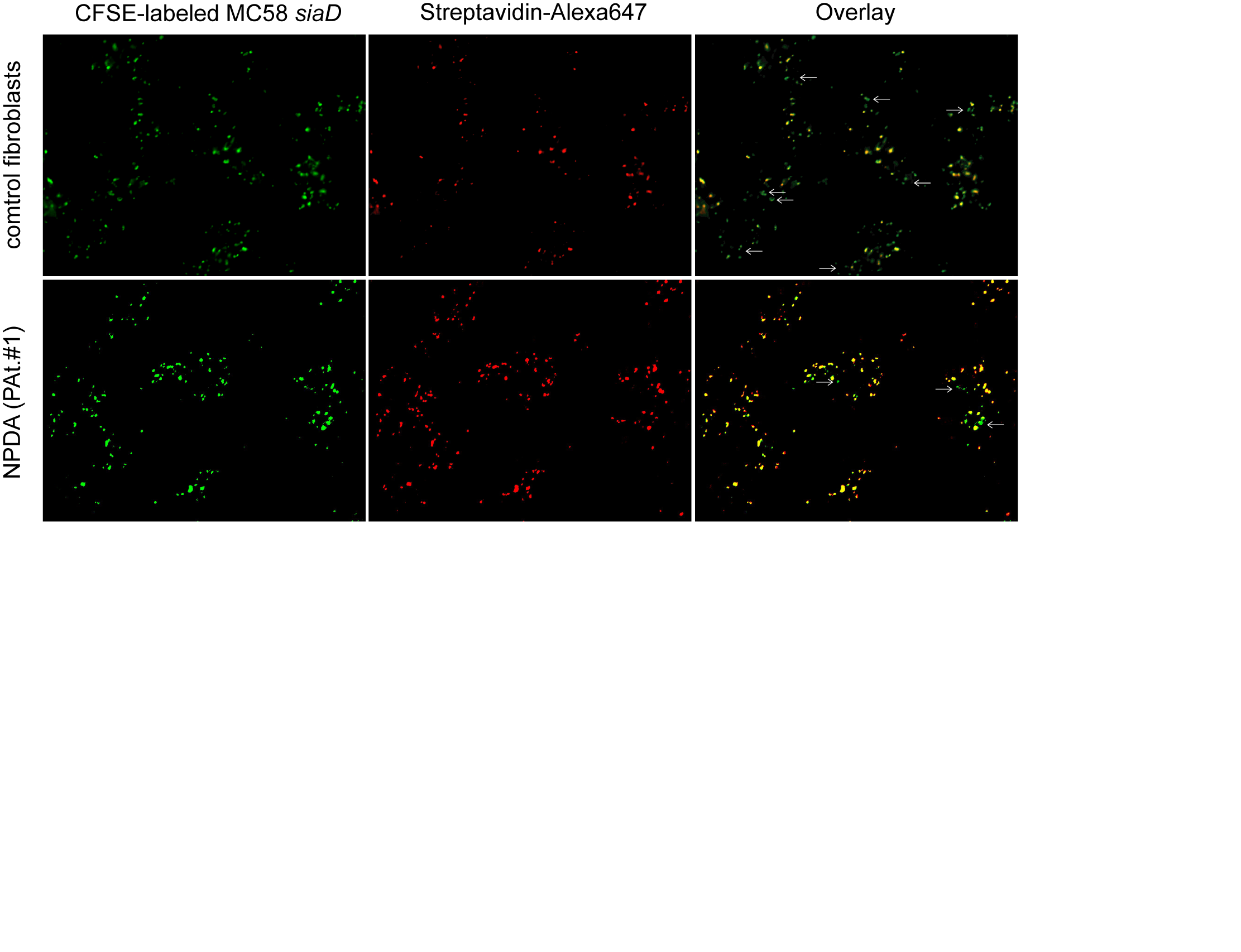

Supplement: Figure S5 — Immunofluorescence of Niemann-Pick disease (NPDA) fibroblasts infected with N. meningitidis . Lower magnification image of NPDA (Pat. #1) and healthy controls fibroblasts infected with CFSE-labeled N. meningitidis strain MC58 siaD at 4 hrs p.i. Extracellular bacteria stain positive with both Streptavidin-Alexa647 (red fluorescence) and CFSE (green fluorescence), whereas intracellular bacteria (arrows) are labeled with CSFE only. (TIF) [file ppat.1004160.s005.tif]

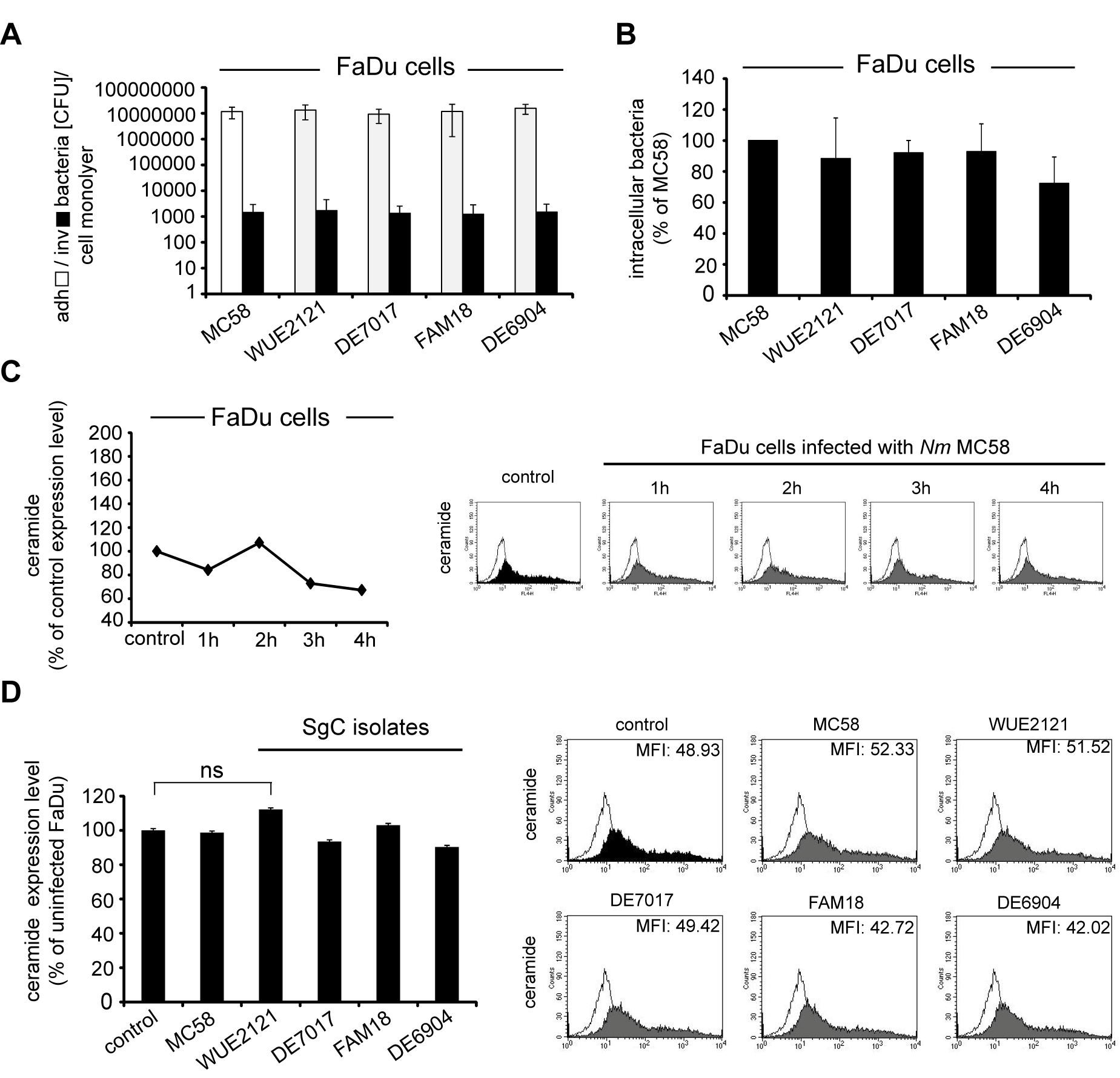

Supplement: Figure S6 — Estimation of adherence and invasion of N. meningitidis strains to FaDu cells and release of ceramide. (A) FaDu cells were infected with strain MC58 and four MenC strains (WUE2121, DE7017, FAM18, DE6904) for 4 hrs p. i. and the number of adherent and invasive bacteria was estimated. The graphs represent mean value ± S.D. of three different independent experiments done in duplicate. * P<0.05. (B) Relative amount of intracellular bacteria compared to strain MC58. (C) FaDu cells were infected with strain MC58 for a 4 hrs time period and surface ceramide release was determined at indicated time points by flow cytometry. Representative FACS analyses on FaDu cells infected with strain MC58, control, uninfected FaDu. Open histograms indicate isotype control, filled histograms show ceramide staining. (D) Surface ceramide release on FaDu cells that were either left uninfected (control) or were infected with MC58 and four MenC strains (WUE2121, DE7017, FAM18, DE6904) was determined by flow cytometry. Open histograms indicate isotype control, filled histograms show ceramide staining. All data show mean values ± S.D. of three independent experiments done in duplicate. ns = not significant. (TIF) [file ppat.1004160.s006.tif]

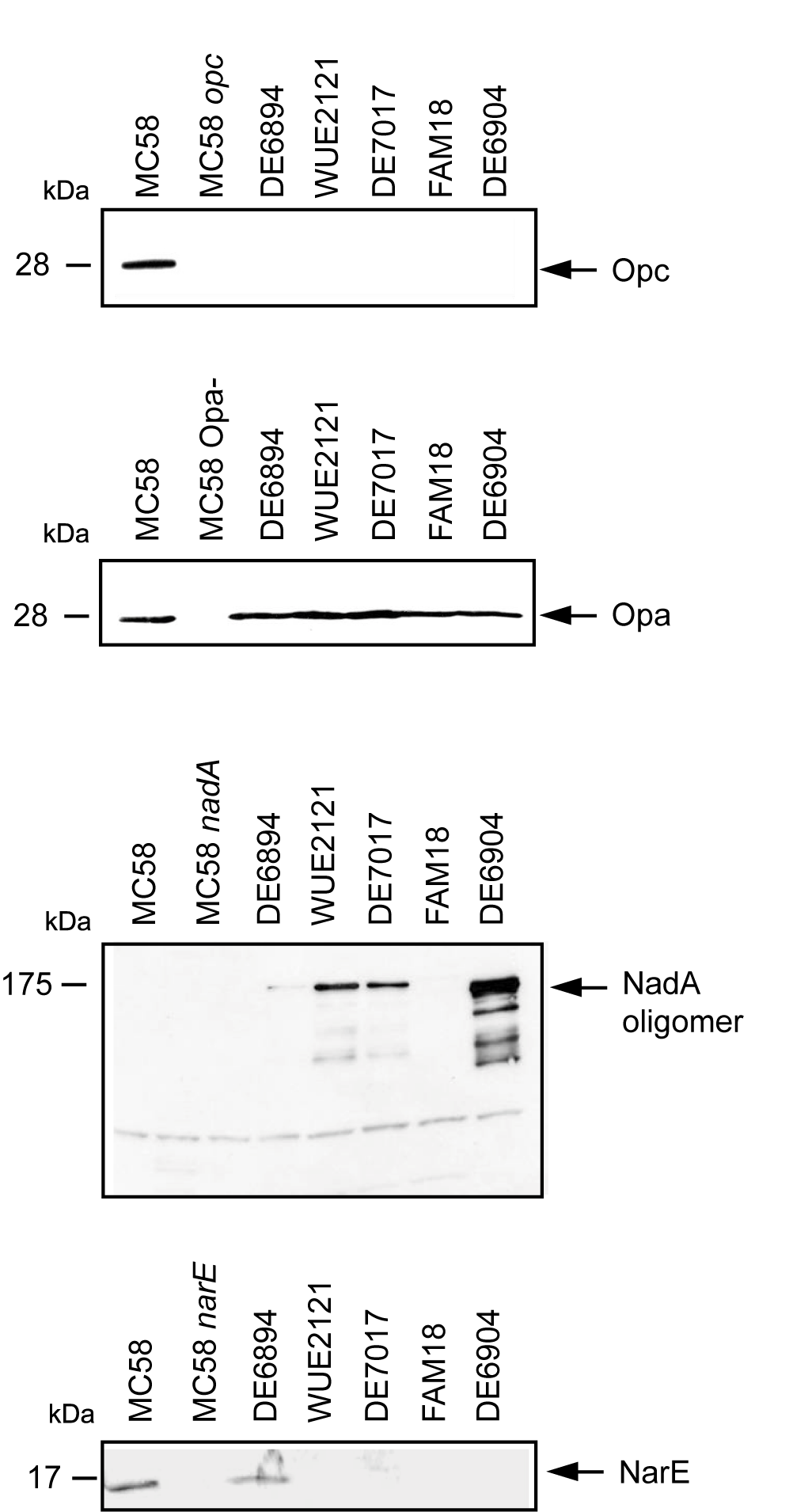

Supplement: Figure S7 — Characterization of expression of adhesins of MenC strains used in this study. Western blot analysis of Opc, Opa, NadA and NarE expression of whole bacterial lysates: MC58, DE6894, WUE2121, DE7017, FAM18, DE6904. As a negative control appropriate isogenic knock out mutants were included: MC58 opc, MC58 Opa-, MC58 nadA and MC58 narE. (TIF) [file ppat.1004160.s007.tif]
